# Percent framing attenuates the magnitude effect in a preference-matching task of intertemporal choice

**DOI:** 10.1371/journal.pone.0262620

**Published:** 2022-01-24

**Authors:** Farid Anvari, Dorina-Diana Verdeș, Davide Marchiori

**Affiliations:** 1 Social and Economic Cognition III, Social Cognition Center Cologne, Faculty of Human Sciences, University of Cologne, Cologne, Germany; 2 Strategic Organization Design Group, Department of Marketing and Management, University of Southern Denmark, Odense, Denmark; Temple University, UNITED STATES

## Abstract

Research in intertemporal decisions shows that people value future gains less than equivalent but immediate gains by a factor known as the discount rate (i.e., people want a premium for waiting to receive a reward). A robust phenomenon in intertemporal decisions is the finding that the discount rate is larger for small gains than for large gains, termed the magnitude effect. However, the psychological underpinnings of this effect are not yet fully understood. One explanation proposes that intertemporal choices are driven by comparisons of features of the present and future choice options (e.g., information on rewards). According to this explanation, the hypothesis is that the magnitude effect is stronger when the absolute difference between present and future rewards is emphasized, compared to when their relative difference is emphasized. However, this hypothesis has only been tested using one task (the two-choice paradigm) and only for gains (i.e., not losses). It’s therefore unclear whether the findings that support the hypothesis can be generalized to different methodological paradigms (e.g., preference matching) and to the domain of losses. To address this question, we conducted experiments using the preference-matching method whereby the premium amounts that people could ask for were framed in terms of either currencies (emphasizing absolute differences) or percentages (emphasizing relative differences). We thus tested the robustness of the evidence in support of the hypothesis that percent framing, relative to currency framing, attenuates the magnitude effect in the domain of gains (Studies 1, 2, and 3) and in the domain of losses (Study 1, 3, and 4). The data were heavily skewed and the assumption of equal variances was violated. Therefore, in place of parametric statistical tests, we calculated and interpreted parametric and nonparametric standardized and unstandardized effect size estimates and their confidence intervals. Overall, the results support the hypothesis.

## Introduction

Intertemporal choices are those in which we make tradeoffs between time and gains (or losses). They typically involve deciding between smaller gains (the principal amount), sooner or in the present, and larger gains (the principal amount plus some premium), later. One descriptive feature of intertemporal choice is the robust finding that people value future gains less than equivalent but immediate gains by a factor known as the discount rate—that is, people want a premium for waiting to receive the gain. The discount rate, although with large individual differences, has been found to be larger for small financial gains than for large financial gains [1]—people ask for larger premiums, relative to the principal amount, to wait for small gains as compared to large gains. For example, someone might prefer £15 now rather than £30 in 3 months but be willing to wait 3 months for £3,000 rather than take £1,500 now, even though waiting doubles the gain in both cases. This phenomenon, termed the magnitude effect, has been replicated across many different elicitation methods (e.g., binary choice and preference matching), samples (e.g., U.S. college students and representative adults from Denmark), contexts (e.g., hypothetical and with real stakes), and framings (e.g., with no context and as financial investments) [2–17]. Various mathematical models have been proposed to describe the pattern of the magnitude effect, but the psychological underpinnings of the phenomenon have received far less attention [11].

Intertemporal choices typically involve two options (e.g., accept money now or wait for a larger amount in the future) which can be considered as consisting of features of the two options (i.e., the present and future amounts) that people can attend to. Such features may include the absolute difference between the amount now and the amount in the future, and the relative difference between the two amounts, whether as interest rates or as proportional differences. We suggest that people compare the two options based on the salient features that are attended to and that they use these comparisons to guide their decisions [16, 18]. Therefore, how the options are framed, how they are presented to participants, will influence which features are attended to and which are not, thereby influencing participants’ preferences and, consequently, the magnitude effect.

When the same information about the available options is presented to people, the standard economic model of intertemporal choice yields the same prediction regardless of how the options are framed. More generally, the predictions of the economic model, in contrast with several experimental findings [see 19, for a review], are not affected by any kind of information irrelevant to the choice problem, such as the framing of options and of their features. In contrast, models that assume that people base their decisions on the salient features of the options have the advantage of being able to capture differences in behaviour caused by framing the options differently. Under such models, we would expect that the size of the magnitude effect will also be impacted by varying the way the options are framed. Specifically, we argue that options can be framed in such a way that attention is drawn to either the absolute or relative (proportional) differences between the amounts involved. We hypothesized that the magnitude effect will be stronger when absolute differences are emphasized compared to when relative differences are. Indeed, this hypothesis has been proposed by, and embedded in, heuristic models of intertemporal choice, such as the DRIFT [16] and the ITCH [18] models.

According to these heuristic models of intertemporal choice, the available options have features which can be made to be less or more salient to the decision maker by manipulating how the options are framed. Under this framework, the relative salience of a feature determines how much it contributes to the option’s overall value or attractiveness. Thus, how the options are framed can direct the decision maker’s attention to some features more than others, changing the relative importance of the features. For ease of reference, in this paper, we focus on the first two features of DRIFT: (1) the absolute difference between options (i.e., the difference between the amount now and the amount in the future—the premium needed to wait in absolute terms); and (2) the relative or proportional difference between options (i.e., the difference between the amount now and the amount in the future, divided by the amount now—the premium needed to wait relative to the principal amount). The ITCH model analogously holds that options are compared in both absolute and relative terms, and that the results of comparisons are additionally aggregated via decision weights. For a full account of the DRIFT and ITCH models, we recommend reading [16] and [18].

Because not all features can be attended to fully, given that people have limited attentional capacity, attention on one feature reduces attention given to another. The options can be framed to emphasize some features and/or de-emphasize others, changing the salience of these features, and thereby impacting people’s decisions. All of this happens without altering the accuracy and amount of information provided to the decision maker. That is, the differently framed decision scenarios are equivalent from an economic perspective. Therefore, whereas from an economic perspective there should be no impact of framing, DRIFT—as well as the ITCH model [18]—predicts that framing can influence people’s preferences in financial intertemporal choices.

Most intertemporal choice studies adopt methods in which the framing directs people’s attention to the absolute difference between the options [16]. For example, studies commonly ask people to choose between an amount now and a larger amount in the future or, alternatively, an amount now and that same amount plus a bonus amount in the future. With the preference matching elicitation method, studies typically ask participants how much money later would be just the same as some amount now [1, 5, 6, 12, 20] so that participants have to think about how much of a bonus would make waiting to receive the money acceptable. Given that such framing tends to emphasize the absolute difference between the two amounts (i.e., the additional earnings), the magnitude effect can be explained, at least partly, by the fact that the same bonus amount equates to a greater discount rate for smaller principal amounts than for larger principal amounts. For example, take the principal amounts of £10 and £100, plus a bonus amount of £10 for waiting for either (i.e., £10 now vs £20 later, and £100 now vs £110 later). This same bonus for the two principal amounts equates to a larger discount rate, or larger premium in relative terms (i.e., a percentage premium), for the smaller principal: the percentage premium for the small principal is 100% and the percentage premium for the larger principal is 10%.

In contrast, intertemporal choices can be framed such that relative differences between the options are emphasized (the second feature of DRIFT). When people attend to the relative differences between the two options, the magnitude effect will be reduced because attention is taken away from the absolute difference [16, 18]. For example, in the choice between £10 now (small principal) and £15 later the absolute difference is £5, whereas when the choice is between £20 now (large principal) and £30 later the absolute difference is £10. When the absolute difference is emphasized, people find the later option in the second pair more attractive, as compared to the later option in the first pair, given the larger absolute additional earnings. However, when presented as a percentage relative to the principal, both pairings provide an additional 50% gains for waiting, reducing the perceived discrepancy between the additional gains for the small and large principal amounts. That is, when relative differences are emphasized, the later options in both pairs seem more similar to each other in magnitude. This reduces the discrepancy in discount rates between the small and large principal amounts and thus reduces the magnitude effect.

Taken together, the DRIFT and ITCH models propose an important and insightful hypothesis for intertemporal choice. Namely, when choices are framed to emphasize relative differences between the options, deemphasizing absolute differences, the magnitude effect will be attenuated (the attenuation hypothesis). Although the DRIFT and ITCH model explanations for the magnitude effect seem promising, experimental evidence for the attenuation hypothesis is limited in two main ways.

First, the hypothesis has only been tested using the binary choice task [16, 18]. According to the binary-choice paradigm, participants are asked to choose between two options—each of the type (x_i_, t_i_), i = 1, 2—that differ in the size of the reward (x_i_) and the time of delivery (t_i_). This leaves open the question about whether the results supporting the attenuation hypothesis are due to idiosyncratic qualities of binary choice tasks. Indeed, different methods for eliciting intertemporal preferences can produce substantially different results [12, 21, 22]. [12] showed that the binary choice method can elicit higher as well as lower discount rates, compared to preference matching, depending on the range of outcomes presented to participants—such tasks may bias participants’ responses by implicitly suggesting appropriate discount rates [19]. In contrast, the comparative process of present and future options in preference-matching is implicit and unconstrained: Participants are required to think about different bonus amounts they would be willing to wait for, thereby simply comparing the future amount they have in mind with the amount presented to them.

Both elicitation methods are associated with advantages and disadvantages [12]. Preference matching introduces less experimenter bias, minimizes experimental demand effects, and provides a more accurate estimation of participants’ indifference points. On the other hand, the binary choice method generally outperforms the preference matching method in predicting self-reported real-world behaviour and outcomes [12]. Notwithstanding this latter point, if the psychological mechanism posited by DRIFT and ITCH is correct, we should observe an attenuation of the magnitude effect not just in binary choice tasks, but also in preference matching, when the options are framed to emphasizes the difference between them in percentage points. If we do not observe such an attenuation, then the DRIFT and ITCH models’ mechanism is less likely to be correct, regardless of how well choice-based tasks predict real-world behaviours or outcomes.

Moreover, from a broader methodological point of view, research shows that study results are often contingent on the design choices of the researchers [23], and that they may not be generalizable to other stimuli [24–27]. Therefore, the robustness and generalizability of the hypothesis that framing options to emphasize relative differences (or deemphasize absolute differences) attenuates the magnitude effect needs to be examined with different research designs and contexts. We do this by using an elicitation method that does not involve direct comparison of existing options but instead requires participants to spontaneously formulate their own preferences. If we observe that when the choice is framed to emphasize relative differences, compared to when framed to emphasize absolute differences, the magnitude effect is attenuated, this evidence would strengthen support for the DRIFT and ITCH models in intertemporal choice, e.g., [16, 18]. However, if we do not find evidence to support the hypothesis, this may indicate that the findings in the literature may be constrained, not generalizable to other elicitation methods [28].

The second main limitation of current evidence for the DRIFT and ITCH models more generally, and the attenuation of the magnitude effect hypothesis more specifically, is that it has only been examined in the domain of gains. Intertemporal preferences may also be elicited for losses (e.g., paying a smaller fine, sooner, versus paying a larger fine, later), but there is less research on the magnitude effect in the loss domain than in the domain of gains. For a detailed explanation of the differences and similarities between intertemporal choice in the loss and gain domains, we refer readers to [11]. Whereas for gains a high discount rate, or a high percentage premium relative to the principal amount, reflects a desire for a sooner gain, for losses it reflects a desire to postpone the loss [11]. Research suggests that, although discount rates for losses are typically smaller than for gains, a magnitude effect can also be observed in the domain of losses [1, 5, 9, 29]; and the magnitude effect may be smaller for losses than for gains [2]. Nevertheless, following the same reasoning as provided for the domain of gains, if people compare the features of options to make their choice, as proposed by the DRIFT and ITCH models, then we would expect that framing options in a way that emphasizes relative differences, compared to framing that emphasizes absolute differences, will attenuate the magnitude effect. Indeed, DRIFT and ITCH make no distinction between gains or losses. Given that current evidence in support of the attenuation hypothesis is limited to the domain of gains, e.g., [16, 18], our experimental investigation in the domain of losses makes an important contribution to the literature.

Taken together, current evidence for the attenuation hypothesis is limited to only one methodological paradigm—the binary choice task—and to the domain of gains, leaving open the possibility that these findings may stem largely from homogenous research designs. The generalizability of these findings is thus in question. To address this, we aimed to examine (1) how framing questions to (de)emphasize absolute or relative differences between the options would impact the size of the magnitude effect in a preference matching task—the method Thaler (1981) used to show the existence of the magnitude effect—and (2) whether the same hypothesis would hold up in the domain of losses. We hypothesized that there would be a magnitude effect (i.e., the percentage premium will be higher for the small principal than for the large principal), but that this magnitude effect would be smaller in the percent frame than in the currency frame—the attenuation hypothesis. We expected this hypothesis to hold in the domain of gains and in the domain of losses.

We pre-registered the methods and sampling procedure for Studies 1, 2, and 3 on the Open Science Framework (https://osf.io/48tza, https://osf.io/9f4ws, https://osf.io/46ycf respectively). We also pre-registered the analysis plan for each of these studies but we had to adjust our analyses for two reasons. First, the data were highly skewed *and* there were unequal variances between the groups. Second, the nonparametric ANOVAs and the nonparametric analysis of simple effects that we pre-registered and originally conducted targeted different statistical variables. The nonparametric ANOVAs were on the adjusted means, whereas the nonparametric simple effects were on the medians. Given these problems with our initial analysis plan, we found an alternative way to conduct the analyses. In this paper, therefore, we report improved analyses that weren’t pre-registered, but which better address the problems inherent to the nature of our data and harmonize the assessment of the overall and simple effects. We report the pre-registered analyses in full in the S1 and S2 Files (see here https://osf.io/qgxpf/). The conclusions remain qualitatively unchanged.

## Methods

We examined the attenuation hypothesis in 4 studies, predicting that the percent frame would attenuate the magnitude effect for both the domain of gains and the domain of losses. We report the methods of each of the studies first, and then report the results together. All studies were conducted online, with participants recruited using the Prolific.co platform (https://www.prolific.co/). We used the same prescreening criteria in all studies (to include only participants with U.K. residency and who were fluent in English) and we prevented participants from participating in more than one study. Protocols for all studies were approved by the Research Ethics Committee of the University of Southern Denmark, approval number 19/71830.

### Study 1

#### Participants

Based on power analyses reported in S2 File (see Methods of Study 1) we aimed for 202 participants to test the interaction hypothesis for the gain domain and an additional 202 participants for the loss domain. After pre-registered exclusions, and keeping only the first entry of 1 participant for whom we had two entries, we recruited a total of 403 participants; 279 female, 121 male, and 3 other, mean age 36.6 years (*SD* = 11.5). Due to the randomization feature of our software there was variability in the number of participants in each condition.

#### Design

Study 1 was an experiment with 8 conditions; a 2 (domain: gains vs losses) by 2 (principal amount: small vs large) by 2 (framing: currency vs percent) mixed design, with domain and framing being between-subjects factors and principal amount a within-subjects factor.

#### Procedure

Participants first answered two preliminary questions, by typing into a text box, to become familiarized with what the task involved and to make sure they understood the questions and were paying attention to the task. Both preliminary questions were presented on the same page and with the same framing and domain as the condition to which participants were randomly allocated. Participants were asked to fill in the blank (italics are for the gain domain and square brackets for the loss domain):

Imagine that someone *is owed* [owes] £100 by the government and they said that they’d feel just the same about *receiving* [paying] the £100 immediately or postponing and *receiving* [paying] a *bonus* [fee] of £50 on top of the £100 later.
Based on this, we can assume that they are indifferent between *receiving* [paying] £100 now and *receiving* [paying] £100 plus a *bonus* [fee] of £ __ later.

The second preliminary question that participants had to answer correctly was worded in exactly the same way but it replaced the £50 bonus/fee with a £200 bonus/fee. For the percent frame, the pound (£) symbol was replaced with the percent symbol (%) appearing after the numerical digits. If participants answered one of these questions incorrectly, they received a warning that they had one attempt left to give correct answers to the preliminary questions. After a second incorrect attempt, they were excluded from participating further.

After the preliminary questions, participants were given the following instructions (italics for gains and square brackets for losses):

Imagine that there was a legitimate error on your back taxes in *your* [the government’s] favor (that is, you paid *more* [less] taxes than you had to), and that you are given two options for *receiving* [paying] your *credit* [debt]. You can have your money *transferred* [withdrawn] immediately *in* [from] your bank account, or in 3 months from now with the addition of a *bonus* [fee].
In the next two scenarios, you will have to indicate HOW MUCH OF A *BONUS* [FEE] (IN BRITISH POUNDS, ON TOP OF THE ORIGINAL AMOUNT) WOULD MAKE *RECEIVING* [PAYING] THE MONEY LATER JUST THE SAME AS *RECEIVING* [PAYING] THE MONEY NOW.

Following these instructions, participants were to answer two questions (presented in random order but both on the same page) by filling in the blanks (italics for gain domain and square brackets for losses):

You *are owed* [owe] £15. How much of a *bonus* [fee] (in £) would make *receiving* [paying] the **£15 + *bonus* [fee]** in 3 months just the same as *receiving* [paying] the £15 now?
I am indifferent between *receiving* [paying] £15 now and *receiving* [paying] £15 plus a *bonus* [fee] of £__ in 3 months.

The second question was exactly the same except that the small principal amount of £15 was replaced with a larger principal amount of £1,500. For participants in the percent frame condition the pounds symbol (£) was replaced with the percent symbol (%) following the text box.

After this task, we asked participants their sex, year of birth, education level, and, optionally, to provide spontaneous comments on the survey.

### Study 2

A small number of participants in Study 1 used the text box at the end of the survey to comment that they found the questions difficult to understand, indicating some confusion. Therefore, we reconsidered the wording of the questions. All methods and procedural details were very similar to Study 1, except that we focused only on the gain domain and collected no data for the loss domain.

#### Participants

In addition to improving the clarity of our questions, we aimed for a larger sample size to increase statistical power for the same effect sizes, though we did not conduct power analyses for this study. We aimed for a total of 260 participants (130 per between-subjects condition). After pre-registered exclusions, we ended with a sample size of 261 participants; 162 female, 97 male, and 2 other, mean age 36.7 years (*SD* = 10.7).

#### Design

Study 2 was an experiment with 4 conditions: a 2 (principal amount: small vs large) by 2 (frame: currency vs percent) mixed design. Principal amount was a within-subjects factor and frame was a between-subjects factor.

#### Procedure

The procedure was exactly the same as for Study 1 except that the wording of the instructions and questions were slightly changed. As in Study 1, the small and large principal questions were presented in random order but on the same page. For the precise details of the wording of the instructions and questions, we refer readers to the S1 File (see under heading, “Study 2 –Procedure & Pre-Registered Dependent Variables”). After completing the intertemporal choice task, we asked participants their sex, year of birth, education level, to “Please rate the clarity of the questions of this survey” (from 1 = *not clear at all*, to 7 = *very clear*, without labelling the points in between; *M* = 5.51, *SD* = 1.70), and an open text box to provide comments on the clarity of the questions. According to the ratings, people seemed to find the questions quite clear.

### Study 3

In Study 3, we examined the attenuation hypothesis in both the domain of gains and the domain of losses. All methods and procedural details were very similar to Studies 1 and 2. We used shorter and more precise instructions (full details provided in the S1 File). In addition, there were two main differences from the previous studies. First, we used different principal amounts: the small principal amount for this study was £45 and the large principal was £1,300. Second, in Study 3 we presented the small and large principal amounts to participants in random order and on separate pages (participants couldn’t go back to change their responses to the earlier question), so that some participants saw the small principal first and some saw the large principal first.

#### Participants

We aimed for a total of 600 participants. After pre-registered exclusions (i.e., those who failed to complete the survey and the second response of those who were recorded twice), we ended with a sample size of 593 participants. Participants were 376 females, 215 males, and 2 other, mean age 36.9 years (*SD* = 13).

#### Design and procedure

Study 3 was an experiment with 8 conditions with an identical design to Study 1. Apart from the wording of the questions, the different principal amounts, and that the principal amounts were presented on separate pages in randomized order, the procedure was the same as Study 1.

### Study 4

All methods and procedural details were identical to Study 3 except we only collected data for the loss domain and the principal amounts were again changed. The small principal amount was £12 and the large principal was £750. Full details are provided in the S1 File.

#### Participants

We aimed for a total of 300 participants. After excluding participants who failed to complete the survey (whose responses were not recorded) and the second response of those who appeared twice in the dataset, we ended with a sample size of 298 participants. Participants were 199 females, 98 males, and 1 other, mean age 37.2 years (*SD* = 13.2).

#### Design and procedure

Study 4 was an experiment with four conditions: a 2 (principal amount: small vs large) by 2 (frame: currency vs percent) mixed design. Principal amount was a within-subjects factor and frame was a between-subjects factor. The procedure was the same as for Study 3.

### Measures

For every study, we took the bonus that people asked for, or the fee that people were willing to pay, in percentage points relative to the principal amount: We refer to this measure as the rate *a*, or the percentage premium. The formula for the percentage premium for participants in the currency frame is a = 100 * Bonus / Principal. The percentage premium for participants in the percent frame is simply their response which reflected the percentage of the principal that they required as a bonus for waiting. For the loss domain the formula is the same except that instead of a “Bonus” it is a “Fee” that’s used.

## Results

Across all studies, our data were extremely skewed, as indicated by visual inspection and Shapiro-Wilk normality tests, which were not sufficiently fixed by log transformations (all *p*s < .001). Moreover, Levene’s tests of homogeneity of variances showed that there were unequal variances between the two principal amounts for some of the comparisons necessary to test for the magnitude effect. For example, in Studies 1, 2, and 3, there were unequal variances between the small and large principal amounts of the currency frame in the gains domain (all *p*s < .01). Similarly, in the losses domain of Studies 3 and 4, the small and large principal amounts of the currency frame had unequal variances (*p* = .0042 and *p* < .0001, respectively). Whereas log transforming the percentage premiums (see below) reduced the unequal variances for the gains domain, the issue of unequal variances remained for the large principal amounts of the losses domain in Studies 3 and 4 (*p* = .0002 and *p* < .0001, respectively). The combination of nonnormality and unequal variances creates problems for the both parametric and nonparametric significance tests. Consequently, adopting a metanalytical perspective, we calculated different effect size metrics reflecting the size of the magnitude effect for the different framing conditions and use these to interpret the direction and magnitude of these effect sizes without resorting to statistical significance tests. To do this, for each participant, we subtracted the percentage premium for the large principal amount from the percentage premium for the small principal amount. This difference score in percentage premiums reflects the size of the magnitude effect for each person. We then calculated the mean and median of the individual magnitude effects for each of the framing conditions.

For each framing condition, we thus report and compare (1) the mean magnitude effect, (2) the corresponding standardized effect size estimate of this mean, Cohen’s *d*, and (3) the median magnitude effect. In addition, we used the Wilcoxon signed-rank test (via the wilcox_effsize() function from the “rstatix” R package [30]) to calculate (4) the standardized nonparametric effect size of the magnitude effect, *r*, in each framing condition. Finally, as another way of addressing skew and unequal variances, we log-transformed the percentage premium, *a*, to obtain *log*.*a*. To avoid uncontrolled data loss, before log-transforming the percentage premium we added 1 (i.e., +1) to the percentage premium of every participant. We then used the *log*.*a* variable to calculate and report the above four effect size estimates of the magnitude effect separately for the currency and percent conditions. R code for all analyses can be found on the Open Science Framework at https://osf.io/qgxpf/.

### Main results

Figs A1–A4 in S2 Appendix show the distribution of responses of the percentage premium, *a*, for the small and large principal amounts across the currency and percent conditions in Studies 1–4, respectively. Table 1 presents the effect size estimates of the magnitude effect for the currency and percent framing conditions in Studies 1–4. For ease of readability, Table 1 only includes the parametric effect sizes (i.e., mean differences and Cohen’s *d*) on the untransformed and transformed percentage premiums. Table A1 in S1 Appendix includes the nonparametric effect sizes. The top panel shows the effect sizes for the gains domain and the bottom panel shows the effect sizes for the losses domain. Positive values reflect a magnitude effect, and the larger the value the larger the magnitude effect. As can be seen in Table 1 (and Table A1 in S1 Appendix), no matter how the effect size is calculated, and no matter whether we use *a* or *log*.*a*, the magnitude effect in the currency frame is *always* larger than the magnitude effect in the percent frame, for both gains and losses. The results of Studies 1–4 therefore provide consistent support for the hypothesis that the percent frame attenuates the magnitude effect.

**Table 1:** Main results. The size of the magnitude effect in each Study [and 95% confidence intervals], partitioned by condition and presented as unstandardized mean differences and as standardized effect sizes Cohen’s *d*, for the untransformed (‘*a*’) and transformed (‘*log*.*a*’) outcome variable.

**GAINS**						
		*a*		*log*.*a*		
Study	Framing	Mean	Cohen’s *d*		Mean	Cohen’s *d*
Study 1	Currency *n* = 99	43.41 [25.05, 61.76]	0.49 [0.26, 0.68]		0.87 [0.66, 1.07]	0.59 [0.55, 1.13]
	Percent *n* = 98	32.39 [-28.08, 92.85]	0.15 [-0.09, 0.31]		0.53 [0.24, 0.81]	0.33 [0.16, 0.57]
Study 2	Currency *n* = 129	71.11 [48.30, 93.92]	0.61 [0.36, 0.73]		0.93 [0.75, 1.11]	0.82 [0.63, 1.14]
	Percent *n* = 132	33.06 [2.86, 63.25]	0.26 [0.02, 0.36]		0.54 [0.30, 0.77]	0.36 [0.22, 0.57]
Study 3	Currency *n* = 155	40.25 [28.02, 52.48]	0.56 [0.36, 0.69]		0.80 [0.61, 0.98]	0.66 [0.44, 0.90]
	Percent *n* = 147	7.69 [-3.41, 18.78]	0.15 [-0.05, 0.28]		0.30 [0.08, 0.51]	0.21 [0.06, 0.39]
**LOSSES**						
		*a*		*log*.*a*		
Study	Framing	Mean	Cohen’s *d*		Mean	Cohen’s *d*
Study 1	Currency *n* = 103	31.25 [0.34, 62.16]	0.28 [0.002, 0.39]		0.85 [0.58, 1.11]	0.53 [0.35, 0.70]
	Percent *n* = 103	6.95 [-5.60, 19.50]	0.13 [-0.09, 0.30]		0.26 [0.05, 0.47]	0.15 [0.04, 0.44]
Study 3	Currency *n* = 154	8.01 [3.96, 12.06]	0.29 [0.15, 0.48]		0.34 [0.14, 0.55]	0.24 [0.11, 0.43]
	Percent *n* = 137	-37.96 [-98.19, 22.27]	-0.15 [-0.28, 0.06]		-0.03 [-0.25, 0.20]	-0.02 [-0.19, 0.15]
Study 4	Currency *n* = 151	21.27 [12.95, 29.58]	0.42 [0.25, 0.58]		0.66 [0.43, 0.89]	0.39 [0.29, 0.63]
	Percent *n* = 147	-5.77 [-21.12, 9.59]	-0.08 [-0.22, 0.10]		-0.03 [-0.25, 0.18]	-0.02 [-0.19, 0.14]

*Note*. a = untransformed percentage premium. log.a = log-transformed percentage premium. Mean = mean of magnitude effect. Positive values reflect a magnitude effect such that the percentage premium for the small principal is larger than for the large principal. The mean is the unstandardized effect size of the magnitude effect, and Cohen’s *d* is the standardized effect size.

### Additional results

Under an alternative explanation, the attenuation of the magnitude effect observed in the losses domain could be a product of a floor effect rather than of framing. Such a floor effect could have affected the large principal amounts in the percent frame, for which participants had very small percentage premiums (see the median of the percentage premium for the large principal amount in the losses domain in the table in S2 File). To rule out this alternative explanation, we removed all participants who gave a response of zero. If the main results in the losses domain were driven by a floor effect, then we should not observe an attenuation effect after removing participants who gave responses of zero, since the analyses would now only include those who were willing to pay some (non-zero) fee. These analyses removed 84 participants from Study 1 (67 from the losses domain and 17 from gains), 15 participants from Study 2 (only gains domain), 152 participants from Study 3 (126 from losses and 26 from gains), and 137 participants from Study 4 (only losses domain). The results of these analyses are presented in Table 2 (Table A2 in the S1 Appendix includes the nonparametric effect sizes). As can be seen in Table 2, the magnitude effect is still always larger in the currency frame than in the percent frame. There is therefore consistent support for the hypothesis that the magnitude effect is attenuated by percent framing, while also ruling out the possibility of a floor effect as an alternative explanation.

**Table 2:** Results after removal of zero premium responses (to rule out floor effects). The size of the magnitude effect in each Study [and 95% confidence intervals], partitioned by condition and presented as unstandardized mean and as standardized effect sizes Cohen’s *d*, in the untransformed (‘*a*’) and transformed (‘*log*.*a’*) outcome variable.

**GAINS**					
		*a*		*log*.*a*	
Study	Framing	Mean	Cohen’s *d*	Mean	Cohen’s *d*
Study 1	Currency *n* = 94	45.82 [26.60, 65.05]	0.51 [0.28, 0.71]	0.94 [0.74, 1.14]	0.78 [0.72, 1.33]
	Percent *n* = 86	35.86 [-33.14, 104.86]	0.16 [-0.10, 0.33]	0.55 [0.26, 0.84]	0.44 [0.18, 0.62]
Study 2	Currency *n* = 126	72.80 [49.53, 96.08]	0.63 [0.37, 0.74]	0.95 [0.77, 1.13]	1.02 [0.68, 1.20]
	Percent *n* = 120	36.32 [3.18, 69.46]	0.27 [0.02, 0.38]	0.65 [0.44, 0.87]	0.56 [0.35, 0.74]
Study 3	Currency *n* = 148	41.53 [28.94, 54.11]	0.57 [0.36, 0.71]	0.83 [0.67, 0.98]	0.82 [0.64, 1.12]
	Percent *n* = 128	9.59 [-2.57, 21.76]	0.18 [-0.04, 0.31]	0.39 [0.21, 0.57]	0.38 [0.20, 0.55]
**LOSSES**					
		*a*		*log*.*a*	
Study	Framing	Mean	Cohen’s *d*	Mean	Cohen’s *d*
Study 1	Currency *n* = 75	43.30 [0.92, 85.69]	0.33 [0.005, 0.47]	1.32 [1.03, 1.61]	1.27 [0.76, 1.45]
	Percent *n* = 64	9.42 [-10.72, 29.56]	0.15 [-0.13, 0.37]	0.34 [0.05, 0.63]	0.26 [0.05, 0.55]
Study 3	Currency *n* = 94	12.13 [6.93, 17.34]	0.37 [0.26, 0.69]	0.72 [0.56, 0.87]	0.70 [0.46, 1.05]
	Percent *n* = 71	-18.87 [-61.17, 23.42]	-0.14 [-0.34, 0.13]	0.19 [-0.07, 0.45]	0.17 [-0.06, 0.41]
Study 4	Currency *n* = 86	32.26 [20.04, 44.48]	0.59 [0.34, 0.80]	1.21 [0.98, 1.43]	1.11 [0.89, 1.54]
	Percent *n* = 75	-10.74 [-41.07, 19.59]	-0.11 [-0.31, 0.15]	0.16 [-0.13, 0.45]	0.13 [-0.10, 0.36]

*Note*. a = untransformed percentage premium. log.a = log-transformed percentage premium. Mean = mean of magnitude effect. Median = median of magnitude effect. Positive values reflect a magnitude effect such that the percentage premium for the small principal is larger than for the large principal. The mean is the unstandardized effect size of the magnitude effect, and Cohen’s *d* is the standardized effect size. The median reflects the unstandardized effect size of the magnitude effect, and *r* is the nonparametric standardized effect size.

There is yet another alternative explanation for the results: people’s tendency (or desire) to be consistent [31–33]. When the questions in preference matching are framed as percentages, it allows people to more easily notice how much more they are discounting the future value of smaller principals compared to the future value of larger principals. In desiring to remain consistent, people can more easily “correct” this inconsistency in the percentage premiums they respond with (i.e., by giving a relatively more similar percentage to both the small and large principal amounts). This would reduce the observed magnitude effect in the precent framing condition.

This alternative explanation could be ruled out by examining the data using only the responses to the principal amount that each participant saw first. Indeed, some people saw the large principal amount first whereas others saw the small principal amount first. We could compare the percentage premiums using only people’s first responses to examine the magnitude effect in a between-subjects analysis. The data in Studies 1 and 2 aren’t suitable for this because participants responded to both principal amounts on the same page. However, in Studies 3 and 4, the small and large principal amounts were displayed sequentially on separate pages of the screen, the order randomized independently for each participant, and participants couldn’t go back to change their responses to the first principal amount they saw. We could therefore examine whether the main results hold while looking only at people’s responses for the first principal amount that they responded to in Studies 3 and 4.

Because we had randomized the presentation order of the principal amounts and the analyses we now report consider only participants’ responses to the principal they saw first, the number of participants across the small and large principal amounts is different than that in the previous analyses. In the gains domain of Study 3 there were 65 and 90 participants in the small and large principal amounts, respectively, for the currency frame; and 63 and 74 participants in the small and large principal amounts, respectively, for the percent frame. In the losses domain of Study 3, there were 76 and 78 participants in the small and large principal amounts, respectively, for the currency frame; and 63 and 74 participants in the small and large principal amounts, respectively, for the percent frame. In Study 4, there were 70 and 81 participants in the small and large principal amounts, respectively, of the currency frame; and 73 and 74 participants in the small and large principal amounts, respectively, of the percent frame.

Examining the magnitude effect in such a between-subjects approach prevented us from creating a difference score reflecting the size of the magnitude effect for each participant. Instead, for each framing condition, we calculated the mean and median of the percentage premium for the small principal and subtracted from this the mean and median, respectively, of the percentage premium for the large principal. We thus have a difference between means and a difference between medians to reflect the between-subjects magnitude effect for each framing condition.

We can thus compare the effect size estimates of the magnitude effect for the currency and percent conditions in terms of the (1) difference between means, (2) the corresponding Cohen’s *d*, and (3) the difference between medians. Like above, we used the Wilcoxon rank sum test to also calculate (4) the nonparametric standardized effect size estimate, *r*. We calculated the four effect size estimates for both the untransformed and transformed percentage premiums in Studies 3 and 4. Table 3 presents the effect sizes only in terms of the mean difference and corresponding Cohen’s *d*. The median differences and nonparametric standardized effect size estimates are presented in Table A2 in S1 Appendix. The results show that the magnitude effect is once again always larger in the currency frame than in the percent frame, ruling out the alternative explanation that the main results were driven by people’s desire to remain consistent.

**Table 3:** Results of between-subjects magnitude effect (looking only at responses to the principal participants saw first). The size of the magnitude effect in each Study [and 95% confidence intervals], partitioned by condition and presented as unstandardized mean differences and as standardized effect sizes Cohen’s *d*, for the untransformed (‘*a*’) and transformed (‘*log*.*a*’) outcome variable.

**GAINS**						
		*a*		*log*.*a*		
Study	Framing	Mean diff	Cohen’s *d*		Mean diff	Cohen’s *d*
Study 3	Currency	59.79 [33.36, 86.22]	0.84 [0.51, 1.17]		1.12 [0.76, 1.47]	1.02 [0.68, 1.36]
	Percent	-2.50 [-18.37, 13.36]	-0.05 [-0.37, 0.27]		0.19 [-0.25, 0.64]	0.14 [-0.18, 0.47]
**LOSSES**						
		*a*		*log*.*a*		
Study	Framing	Mean diff	Cohen’s *d*		Mean diff	Cohen’s *d*
Study 3	Currency	6.65 [0.04, 13.25]	0.32 [0.004, 0.64]		0.40 [-0.04, 0.85]	0.29 [-0.03, 0.61]
	Percent	-24.53 [-65.56, 16.51]	-0.19 [-0.53, 0.15]		-0.21 [-0.70, 0.27]	-0.15 [-0.48, 0.19]
Study 4	Currency	32.47 [14.74, 50.19]	0.63 [0.31, 0.96]		1.21 [0.70, 1.71]	0.80 [0.46, 1.13]
	Percent	-2.85 [-14.39, 8.69]	-0.08 [-0.40, 0.24]		0.20 [-0.30, 0.70]	0.13 [-0.19, 0.46]

*Note*. a = untransformed percentage premium. log.a = log-transformed percentage premium. Mean diff = mean of between-subjects magnitude effect. Positive values reflect a magnitude effect such that the percentage premium for the small principal is larger than for the large principal. The mean diff is the unstandardized effect size of the magnitude effect, and Cohen’s *d* is the standardized effect size.

## General discussion

A robust and widely studied phenomenon in intertemporal choice is the magnitude effect. When deciding between smaller sooner gains and larger later gains, people have a stronger preference for sooner gains, particularly when dealing with smaller sums of money. That is, people require a larger relative premium for waiting to receive smaller financial gains as compared to larger gains. As one promising avenue for developing a psychological understanding for this phenomenon, researchers have proposed psychological models of intertemporal choice and the magnitude effect. We, and others [16, 18], propose that people compare different features of the options available to them in order to help guide their decisions in intertemporal choice tasks. As such, the features of the options that are (de)emphasized will influence what people attend to in making their comparisons and thus their preferences, thereby impacting the observed magnitude effect [16, 18]. We conducted 4 studies that tested a key prediction of these models: that the magnitude effect will be attenuated when relative differences between the present and later rewards or penalties are emphasized, compared to when absolute differences are emphasized. Across all 4 studies, we found that the magnitude effect was smaller when the task had a percent framing (i.e., relative difference emphasized) compared to when the task had currency framing (i.e., absolute difference emphasized), both in the domain of gains and losses.

From an economic perspective, the information provided in the tasks, and the value of the options, remain the same regardless of how the options are framed. From this perspective, therefore, we should have observed no impact of framing on the magnitude effect. However, from a psychological perspective, such as described by the DRIFT and ITCH heuristic models, the perceived values of the options can be impacted by how the options are framed. Our findings showing that the magnitude effect is attenuated by the percent framing are thus consistent with the DRIFT and ITCH models. Specifically, framing options to deemphasize their absolute difference and emphasize their relative difference changes the perceived value of the options such that the magnitude effect is reduced. Our research addressed two gaps in the literature by showing that this attenuation of the magnitude effect is not limited to 1) the binary choice task and 2) to the domain of gains. We found that this attenuation is also observed using the preference matching method and in the domain of losses. We can therefore be more confident in the psychological models that consider the impact of framing on intertemporal choices.

It is worth pointing out that our results in the domain of losses do not necessarily contradict past research showing a reversed magnitude effect [11] but are rather due to methodological differences. [11] found that the magnitude effect in the domain of losses is reversed because they allowed participants to pay a smaller sum in the future, finding that some people preferred to pay a larger amount now than wait and pay a smaller amount later (e.g., pay £10 now or £9 later) such that participants could have negative discount rates. In the present studies, participants could choose to pay zero in fees for waiting but they could not choose to pay less in the future. Nonetheless, in Studies 3 and 4, but not Study 1, there were potential reverse magnitude effects in the percent frame of the losses domain as indicated by the negative sign of the effect sizes in Tables 1 and 2. Thus, it may be the case that people prefer to pay larger debts sooner when the choice is framed such that attention is directed to the relative difference between paying now and paying later. However, we hesitate to draw strong conclusions or interpretations about this finding for two reasons. First, this was not the purpose of the current studies and the finding may be spurious. Second, the effect sizes for a possible reverse magnitude effect had wide confidence intervals that included positive effect sizes. Future research may examine this potential reversal, perhaps with larger sample sizes to obtain more precise estimates. Finally, and in line with past research [2], we found that the magnitude effect was generally smaller for losses than for gains (as can be seen in the Tables reported in the results section).

Future research can be done with a focus on the generalizability of our results, especially for what concerns the time horizon and the presence of real stakes. In particular, the decisions presented to participants in the studies were always hypothetical, without participants experiencing the outcomes of their decisions. Future research may examine the hypotheses using real financial incentives, though this may not be practical with gains of large magnitude and for losses. Moreover, although we used a few different principal amounts across the 4 studies, we did not examine different time horizons. It might also be worth examining whether there are boundary conditions for the attenuation hypothesis with respect to the principal amounts used, particularly in the domain of losses. For example, percent framing may not attenuate the magnitude effect when the principal amounts are relatively larger (e.g., when the small principal amount is £1,500) or it may depend on the proportional size of the large principal amount relative to the small principal amount. Future research can also examine whether the attenuation hypothesis, and the DRIFT and ITCH models more generally, generalize to real-stakes contexts. Does framing the options to (de)emphasize different features impact intertemporal choice decisions when the payoffs are real? It is possible that people will weight features differently for real payoffs and real time delays. Although an examination of this was beyond the scope of the present paper, future research can shed light in this regard.

## Conclusion

Building on research to shed light on the psychological mechanisms that produce the magnitude effect, we tested how variations in the framing of preference matching in intertemporal choice impacts the percentage premium that people ask for (or their discount rates) and, thus, the observed magnitude effect. In line with predictions, and past research, we found that framing influenced people’s discount rates. Specifically, framing the options to emphasize the relative difference between the smaller sooner option and the larger later option attenuated the magnitude effect. Importantly, we not only demonstrated this attenuation in the domain of gains but also for losses, further strengthening support for the attenuation hypothesis and psychological models of intertemporal choice.

## Supporting information

S1 Appendix Tables A1 and A2. Can also be found here https://osf.io/qgxpf/.
(DOCX)

S2 Appendix (DOCX)

S1 File Materials for all studies, including instructions to participants, and results from pre-registered analyses. Can also be found here https://osf.io/qgxpf/.
(DOCX)

S2 File Materials for all studies, including instructions to participants, and results from additional analyses. Can also be found here https://osf.io/qgxpf/.
(DOC)
