# Supplementary material for: Percent framing attenuates the magnitude effect in a preference-matching task of intertemporal choice
Source: PLoS One. 2022 Jan 24;17(1):e0262620. doi: 10.1371/journal.pone.0262620 (PMC8786190; doi:10.1371/journal.pone.0262620)
Supplement: S1 Appendix — Can also be found here https://osf.io/qgxpf/. (DOCX) [file pone.0262620.s001.docx]

# Appendix

Table A1 presents the effect size estimates of the magnitude effect for the currency and percent conditions in terms of the (1) difference between means, (2) the corresponding Cohen’s *d*, (3) the difference between medians, and (4) the standardized nonparametric effect size, *r*. Regardless of which effect size metric we focus on, the percent frame always has a smaller magnitude effect than the currency frame. Table A2 shows that the results hold even when we use only the responses to the principal amount that each participant saw first, ruling out the idea that memory effects were driving the results.

**Table A1. Main results. The size of the magnitude effect in each Study [and 95% confidence intervals], partitioned by condition and presented as unstandardized mean and median differences and as standardized effect sizes Cohen’s d and r, for the untransformed (‘a’) and transformed (‘log.a’) outcome variable.**

| **GAINS** | | | | | | | | | |
| --- | --- | --- | --- | --- | --- | --- | --- | --- | --- |
|  |  | *a* | | | | *log.a* | | | |
| Study | Framing | Mean | Cohen’s *d* | Median | *r* | Mean | Cohen’s *d* | Median | *r* |
| Study 1 | Currency  *n* = 99 | 43.41  [25.05, 61.76] | 0.49  [0.26, 0.68] | 23.33  [14.67, 36.67] | 0.68  [0.55, 0.78] | 0.87  [0.66, 1.07] | 0.59  [0.55, 1.13] | 0.91  [0.69, 1.25] | 0.69  [0.55, 0.79] |
|  | Percent  *n* = 98 | 32.39  [-28.08, 92.85] | 0.15  [-0.09, 0.31] | 0.00  [-11.0, 0.00] | 0.31  [0.12, 0.48] | 0.53  [0.24, 0.81] | 0.33  [0.16, 0.57] | 0.00  [-0.68, 0.00] | 0.35  [0.17, 0.52] |
| Study 2 | Currency  *n* = 129 | 71.11  [48.30, 93.92] | 0.61  [0.36, 0.73] | 33.33  [16.67, 43.33] | 0.67  [0.56, 0.77] | 0.93  [0.75, 1.11] | 0.82  [0.63, 1.14] | 1.08  [1.07, 1.47] | 0.69  [0.58, 0.77] |
|  | Percent  *n* = 132 | 33.06  [2.86, 63.25] | 0.26  [0.02, 0.36] | 19.00  [8.00, 38.00] | 0.49  [0.34, 0.62] | 0.54  [0.30, 0.77] | 0.36  [0.22, 0.57] | 0.67  [0.56, 1.35] | 0.43  [0.28, 0.57] |
| Study 3 | Currency  *n* = 155 | 40.25  [28.02, 52.48] | 0.56  [0.36, 0.69] | 17.22  [8.80, 24.18] | 0.64  [0.53, 0.74] | 0.80  [0.61, 0.98] | 0.66  [0.44, 0.90] | 0.75  [0.55, 0.88] | 0.66  [0.54, 0.75] |
|  | Percent  *n* = 147 | 7.69  [-3.41, 18.78] | 0.15  [-0.05, 0.28] | 5.00  [0.00, 10.00] | 0.41  [0.25, 0.55] | 0.30  [0.08, 0.51] | 0.21  [0.06, 0.39] | 0.39  [0.17, 0.78] | 0.37  [0.21, 0.51] |
| **LOSSES** | | | | | | | | | |
|  |  | *a* | | | | *log.a* | | | |
| Study | Framing | Mean | Cohen’s *d* | Median | *r* | Mean | Cohen’s *d* | Median | *r* |
| Study 1 | Currency  *n* = 103 | 31.25  [0.34, 62.16] | 0.28  [0.002, 0.39] | 6.00  [-1.33, 9.33] | 0.58  [0.43, 0.70] | 0.85  [0.58, 1.11] | 0.53  [0.35, 0.70] | 0.87  [0.57, 1.69] | 0.56  [0.42, 0.68] |
|  | Percent  *n* = 103 | 6.95  [-5.60, 19.50] | 0.13  [-0.09, 0.30] | 0.00  [0.00, 0.00] | 0.21  [0.04, 0.38] | 0.26  [0.05, 0.47] | 0.15  [0.04, 0.44] | 0.00  [0.00, 0.00] | 0.20  [0.02, 0.38] |
| Study 3 | Currency  *n* = 154 | 8.01  [3.96, 12.06] | 0.29  [0.15, 0.48] | 0.90  [-1.55, 1.80] | 0.40  [0.25, 0.52] | 0.34  [0.14, 0.55] | 0.24  [0.11, 0.43] | 0.13  [-0.09, 0.26] | 0.34  [0.20, 0.48] |
|  | Percent  *n* = 137 | -37.96  [-98.19, 22.27] | -0.15  [-0.28, 0.06] | 0.00  [0.00, 0.00] | 0.09  [0.005, 0.26] | -0.03  [-0.25, 0.20] | -0.02  [-0.19, 0.15] | 0.00  [0.00, 0.00] | 0.05  [0.002, 0.21] |
| Study 4 | Currency  *n* = 151 | 21.27  [12.95, 29.58] | 0.42  [0.25, 0.58] | 2.83  [-1.33, 5.67] | 0.53  [0.40, 0.63] | 0.66  [0.43, 0.89] | 0.39  [0.29, 0.63] | 0.20  [-0.37, 0.39] | 0.43  [0.29, 0.57] |
|  | Percent  *n* = 147 | -5.77  [-21.12, 9.59] | -0.08  [-0.22, 0.10] | 0.00  [0.00, 0.00] | 0.06  [0.003, 0.21] | -0.03  [-0.25, 0.18] | -0.02  [-0.19, 0.14] | 0.00  [0.00, 0.00] | 0.006  [0.003, 0.19] |

*Note*. a = untransformed percentage premium. log.a = log-transformed percentage premium. Mean = mean of magnitude effect. Median = median of magnitude effect. Positive values reflect a magnitude effect such that the percentage premium for the small principal is larger than for the large principal. The mean is the unstandardized effect size of the magnitude effect, and Cohen’s *d* is the standardized effect size. The median reflects the unstandardized effect size of the magnitude effect, and *r* is the nonparametric standardized effect size.

**Table A2. Results of between-subjects magnitude effect (looking only at responses to the principal participants saw first). The size of the magnitude effect in each Study [and 95% confidence intervals], partitioned by condition and presented as unstandardized mean and median differences and as standardized effect sizes Cohen’s d and r, for the untransformed (‘a’) and transformed (‘log.a’) outcome variable.**

| **GAINS** | | | | | | | | | |
| --- | --- | --- | --- | --- | --- | --- | --- | --- | --- |
|  |  | *a* | | | | *log.a* | | | |
| Study | Framing | Mean diff | Cohen’s *d* | Median diff | *r* | Mean diff | Cohen’s *d* | Median diff | *r* |
| Study 3 | Currency | 59.79  [33.36, 86.22] | 0.84  [0.51, 1.17] | 36.33  [21.37, 51.28] | 0.47  [0.32, 0.60] | 1.12  [0.76, 1.47] | 1.02  [0.68, 1.36] | 1.03  [0.64, 1.42] | 0.47  [0.33, 0.59] |
|  | Percent | -2.50  [-18.37, 13.36] | -0.05  [-0.37, 0.27] | 10.00  [-5.25, 12.50] | 0.13  [0.01, 0.30] | 0.19  [-0.25, 0.64] | 0.14  [-0.18, 0.47] | 0.39  [-0.21, 0.48] | 0.13  [0.01, 0.30] |
| **LOSSES** | | | | | | | | | |
|  |  | *a* | | | | *log.a* | | | |
| Study | Framing | Mean diff | Cohen’s *d* | Median diff | *r* | Mean diff | Cohen’s *d* | Median diff | *r* |
| Study 3 | Currency | 6.65  [0.04, 13.25] | 0.32  [0.004, 0.64] | 3.63  [-1.62, 9.57] | 0.13  [0.01, 0.28] | 0.40  [-0.04, 0.85] | 0.29  [-0.03, 0.61] | 0.64  [-0.47, 1.52] | 0.13  [0.01, 0.29] |
|  | Percent | -24.53  [-65.56, 16.51] | -0.19  [-0.53, 0.15] | -1.75  [-5.00, 4.00] | 0.05  [0.004, 0.21] | -0.21  [-0.70, 0.27] | -0.15  [-0.48, 0.19] | -0.63  [-1.79, 1.15] | 0.05  [0.003, 0.22] |
| Study 4 | Currency | 32.47  [14.74, 50.19] | 0.63  [0.31, 0.96] | 14.00  [1.67, 19.50] | 0.37  [0.21, 0.52] | 1.21  [0.70, 1.71] | 0.80  [0.46, 1.13] | 1.57  [0.20, 1.96] | 0.37  [0.21, 0.52] |
|  | Percent | -2.85  [-14.39, 8.69] | -0.08  [-0.40, 0.24] | 2.40  [-3.75, 9.75] | 0.08  [0.01, 0.25] | 0.20  [-0.30, 0.70] | 0.13  [-0.19, 0.46] | 0.55  [-1.10, 1.99] | 0.08  [0.01, 0.25] |

*Note*. a = untransformed percentage premium. log.a = log-transformed percentage premium. Mean diff = mean of between-subjects magnitude effect. Median diff = median of between-subjects magnitude effect. Positive values reflect a magnitude effect such that the percentage premium for the small principal is larger than for the large principal. The mean diff is the unstandardized effect size of the magnitude effect, and Cohen’s *d* is the standardized effect size. The median diff reflects the unstandardized effect size of the magnitude effect. The standardized nonparametric effect size is *r*.
