# Supplementary material for: Percent framing attenuates the magnitude effect in a preference-matching task of intertemporal choice
Source: PLoS One. 2022 Jan 24;17(1):e0262620. doi: 10.1371/journal.pone.0262620 (PMC8786190; doi:10.1371/journal.pone.0262620)

**S2 Appendix.**

**Figure A1. Distribution of responses in terms of the percentage premium in Study 1.**

Each dot represents a participant’s response in that condition. Note that for the gain domain there were 3 observations above the y-axis upper limit: 1 from the small principal percent frame, 1 from the large principal percent frame, and 1 from the large principal currency frame. For the loss domain there were 2 observations above the y-axis upper limit: 1 from the large principal percent frame, and 1 from the small principal currency frame.


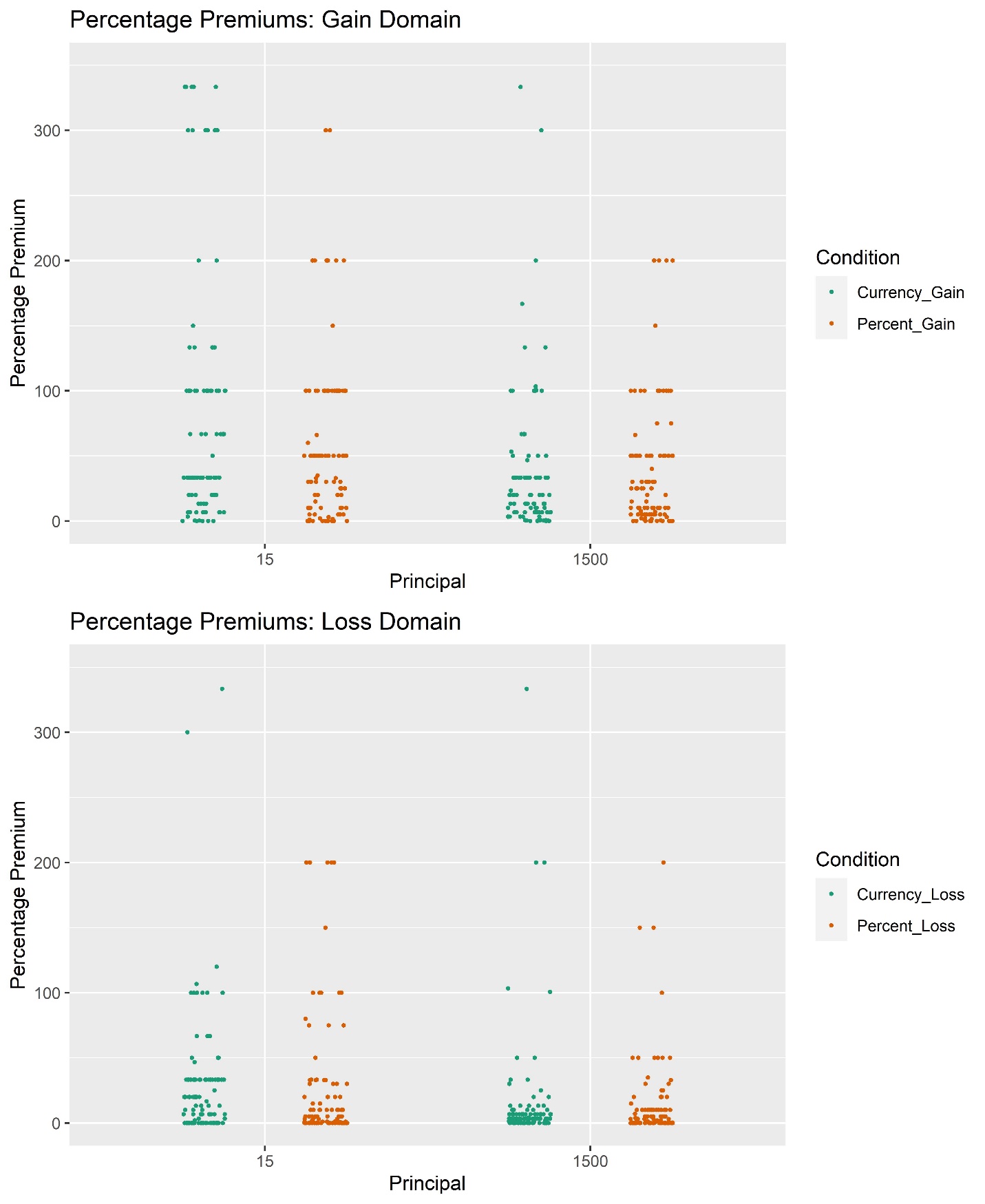


**Figure A2. Distribution of responses in terms of the percentage premium in Study 2.**

Each dot represents a participant’s response in that condition. Note that there were 7 observations fall above the y-axis upper limit: 1 from the large principal percent frame, 3 from the small principal percent frame, and 3 from the small principal currency frame.


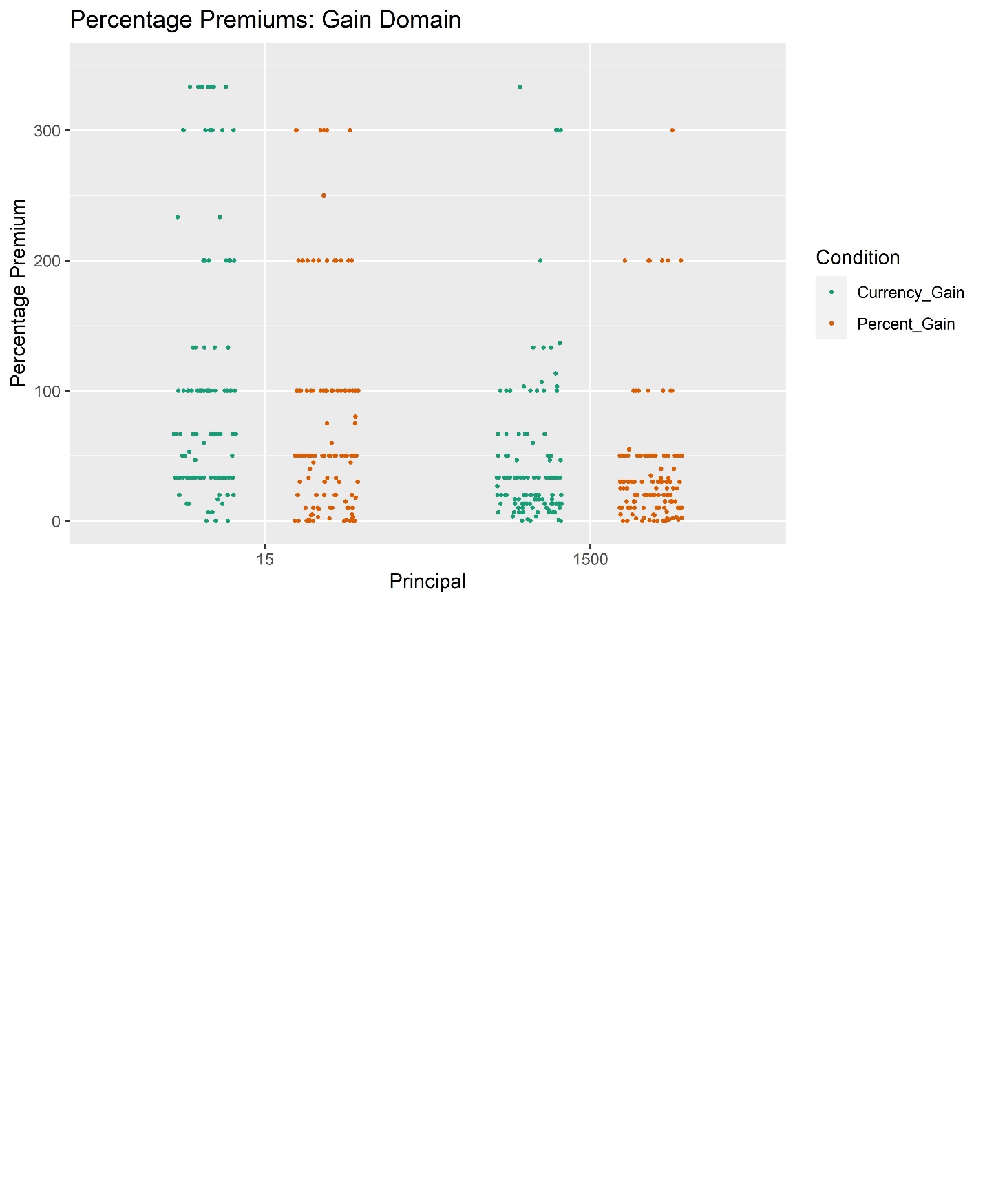


**Figure A3. Distribution of responses in terms of the percentage premium in Study 3.**

Each dot represents a participant’s response in that condition. Note that for the gain domain there were 2 observations above the y-axis upper limit: 1 from the small principal currency frame, 1 from the large principal percent frame. For the loss domain there were 2 observations above the y-axis upper limit: both from the large principal percent frame.


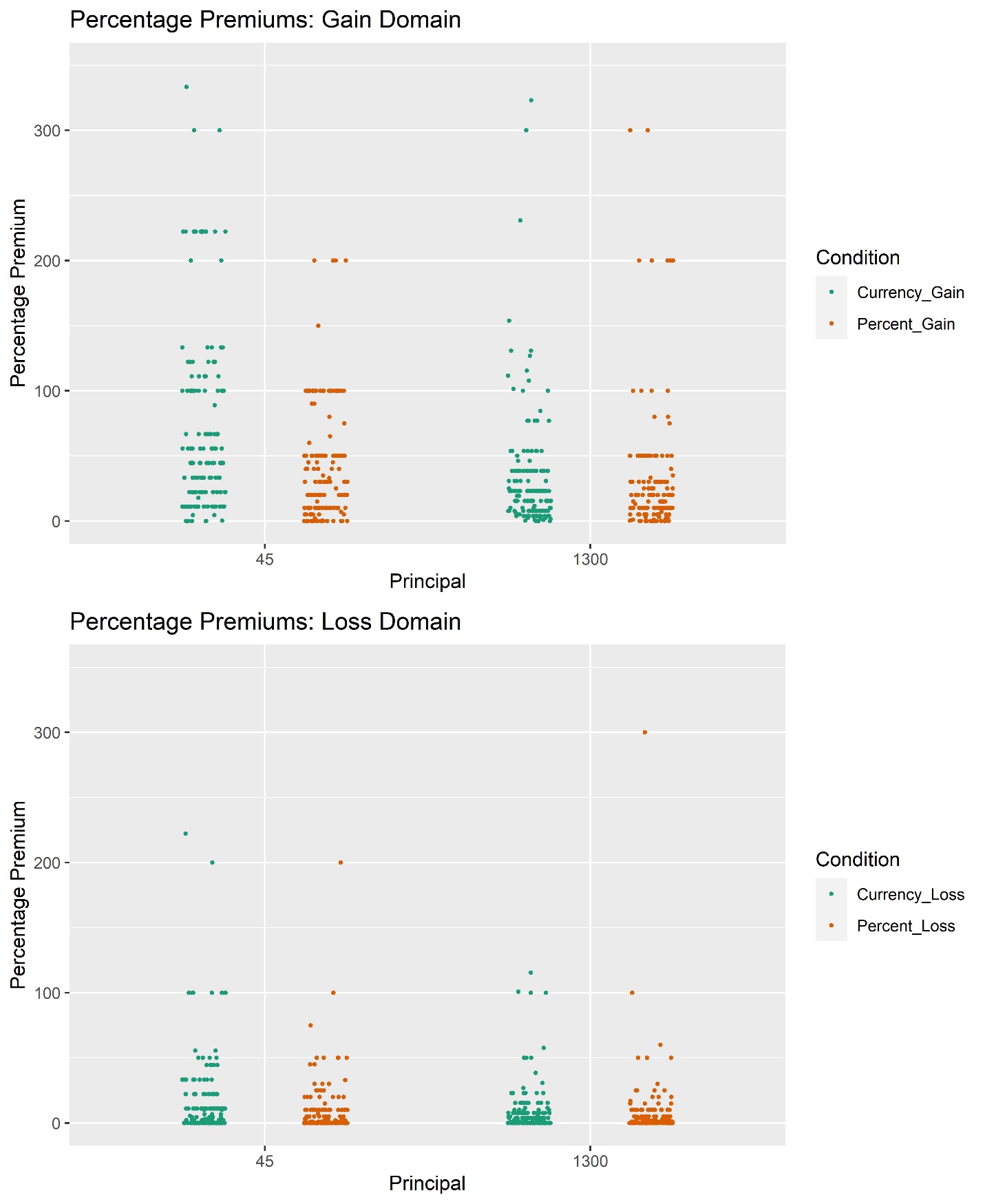


**Figure A4. Distribution of responses in terms of the percentage premium in Study 4.**

Each dot represents a participant’s response in that condition. Note that there were 2 observations above the y-axis upper limit: 1 from the small principal currency frame, 1 from the large principal percent frame.


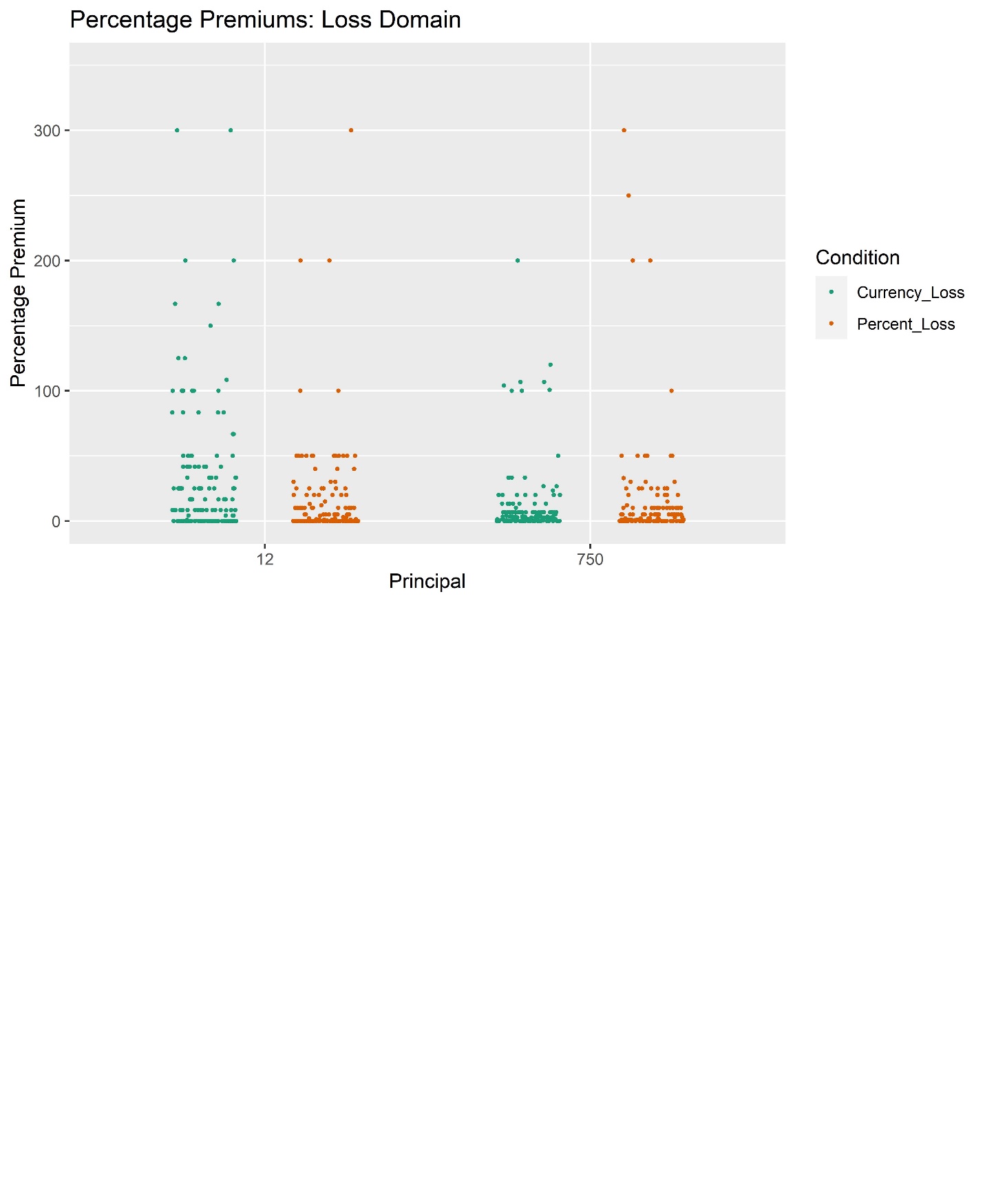

Supplement: S2 Appendix — (DOCX) [file pone.0262620.s002.docx]
