# Supplementary material for: Percent framing attenuates the magnitude effect in a preference-matching task of intertemporal choice
Source: PLoS One. 2022 Jan 24;17(1):e0262620. doi: 10.1371/journal.pone.0262620 (PMC8786190; doi:10.1371/journal.pone.0262620)
Supplement: S1 File — Can also be found here https://osf.io/qgxpf/. (DOCX) [file pone.0262620.s003.docx]

# Study 1 – Pre-Registered dependent variables

Here we report the results of Study 1, first for the parametric ANOVAs on the log-transformed percentage premiums, and then using the dependent variables that we pre-registered.

## Measures

In addition to the percentage premiums reported in the manuscript, the outcome variables we were interested in, as presented in our pre-registration, were people’s exponential and hyperbolic discount rates. These are the two standard non-parametric models of time discount (see Doyle, 2013 for a review).^^[[1]](#footnote-1)^^ According to the model of exponential discounting with continuous compounding (Samuelson, 1937), the present amount P of a future value F is computed as P = F e^- r ∙ T^, where T is the delay time (or delivery time of F, typically expressed in years), and r is the interest rate. For this model, the discount term is thus g(T) = exp (- r ∙ T). According to the hyperbolic discount model (Laibson, 1997), the present value P of a future amount F in T years is given by P = F [1 / (1 + h ∙ T)]; the name of the model follows the hyperbolic form of the discount term f(T) = [1 / (1 + h ∙ T)].

The formulation of the exponential discounting model implies, for example, that given a constant rate r, the ratio g(T + 1) / g(T) = exp(- r) is independent from T. This means that the discount of one unit of time from now (e.g., one month from now) is the same as that of one unit of time from time T (e.g., one month after T = 36 months from now). This is not true for the hyperbolic discount model, for which f(T + 1) / f(T) = 1 – h / (1 + h ∙ T + h), so that (keeping h fixed) the discount of one unit of time is 1 – h / (1 + h) when T = 0, and tends to 1 as T tends to infinite. This property allows the hyperbolic discount model to be able to capture the famous reversal of time preferences observed since Strotz (1955) (also mentioned by Thaler, 1981), which cannot be accounted for by the exponential model.

We estimated, individually for each participant, the discount rates r and h using the models described above. Given the present value P (the principal amount gained/lost in our experimental scenarios, £15 vs £1,500) and the future value F (in our experiment, the sum of the bonus/fee elicited from participants plus the principal amount gained/lost), the discount rates are simply computed as:

1. r = [log(F / P)] / T (Exponential discounting),

2. h = [(F / P) – 1] / T (Hyperbolic discounting),

where T is the delay time expressed in years (in our study the delay time is three months). We used both of these discount rates as dependent variables to check for the generalizability and robustness of our conclusions across the main established theoretical frameworks for calculating discount rates. It is worth noting that in the main text, we adopt as discount rate the rate *a = 100 ∙ Bonus / Principal*: This measure turns out to be a simple rescaling of the rate h defined above, since the future amount F is the sum of the Bonus and the Principal. We do not, however, normalize our rate a by the time delay T, as the variable T is fixed (specifically, T = three months) in our experimental studies.

We pre-registered our intention to use the log transformations of these discount rates in the event that the outcome variables’ distributions substantially deviated from normality, as indeed they did. To correct for the two tests, we decided to apply the Holm-Bonferroni correction for multiple comparisons such that the interaction test with the lowest *p*-value would be tested against an alpha of .025 (i.e., .05 divided by 2 tests) and the other would be tested against an alpha of .05 (i.e., .05 divided by the 1 remaining test). To test the robustness of the results, we also pre-registered running the non-parametric Aligned Rank Transform on the (untransformed) discount rates, which for brevity we refer to as non-parametric ANOVA. A description of the Aligned Rank Transform procedure is given by Wobbrocket al. (2011), and is implemented in the ARTool package for R.

## Results

### Gain Domain

In log-transforming the outcome variables for the gain domain, we lost data from 17 participants for the parametric ANOVAs due to having percentage premiums (and exponential and hyperbolic discount rates) of zero, for which the log-transformation is not defined. Of these 17 participants, in the currency frame, 4 asked for 0 percentage premium for both small and large principal amounts, and 1 asked for 0 only for the small principal (nobody asked for 0 only for the large principal); and in the percent frame, 9 asked for 0 in response to both principal amounts, 1 asked for 0 only for the small principal, and 2 only for the large principal.

First, we report the parametric ANOVA results for the log-transformed percentage premium. The parametric ANOVAs showed that there was an overall magnitude effect such that the (log-transformed) percentage premiums were greater for the small principal than for the large principal (see Table 1 for descriptive statistics), as indicated by the significant main effect of the principal factor, *F*(1,178) = 72.69, *p* < .001. This magnitude effect was qualified by a statistically significant interaction between principal amount and frame, *F*(1,178) = 5.90, *p* = .0161. We used paired-samples *t*-tests, with Bonferroni corrections (i.e., corrected alpha set to .025, to correct for a total of 2 *t*-tests), to examine the pattern of the interactions. As can be seen in Table 1 the magnitude effect—the higher percentage premium for the small principal compared to the larger principal—was greater for participants in the currency frame, *t*(93) = 9.11, *p* < .001, *d* = 0.68, *CI_95%_*[0.52, 0.85], than for participants in the percent frame, *t*(85) = 3.75, *p* < .001, *d* = 0.44, *CI_95%_*[0.20, 0.68]. In sum, the parametric ANOVAs on the log-transformed percentage premiums showed support for the hypothesis: The percent frame attenuated the magnitude effect as compared to the currency frame.

Second, we report results for the exponential and hyperbolic discount rates. The parametric ANOVAs showed that there was an overall magnitude effect such that the discount rate was greater for the small principal than for the large principal (see Table S1 for descriptive statistics), as indicated by the significant main effects of the principal factor, *F*(1,178) = 75.73, *p* < .001 (log-transformed exponential discount rate) and *F*(1,178) = 72.69, *p* < .001 (log-transformed hyperbolic discount rate). This magnitude effect was qualified by statistically significant interactions between principal amount and frame on both the log-transformed exponential discount rate , *F*(1,178) = 6.32, *p* = .0128, and the log-transformed hyperbolic discount rate, *F*(1,178) = 5.90, *p* = .0161. We used paired-samples *t*-tests, with Bonferroni corrections (i.e., corrected alpha set to .0125, to correct for a total of 4 *t*-tests), to examine the pattern of the interactions. As can be seen in Table S1, for exponential discount rates, the magnitude effect was greater for participants in the currency frame, *t*(93) = 8.82, *p* < .001, *d* = 0.65, *CI_95%_*[0.49, 0.81], than for participants in the percent frame, *t*(85) = 3.96, *p* < .001, *d* = 0.44, *CI_95%_*[0.21, 0.67]. Likewise for hyperbolic discount rates: currency frame, *t*(93) = 9.11, *p* < .001, *d* = 0.68, *CI_95%_*[0.52, 0.85]; percent frame, *t*(85) = 3.75, *p* < .001, *d* = 0.44, *CI_95%_*[0.20, 0.68]. In sum, the parametric ANOVAs on the log-transformed exponential and hyperbolic discount rates showed support for Hypothesis 1: The percent frame attenuated the magnitude effect as compared to the currency frame.

The non-parametric ANOVAs on the exponential and hyperbolic discount rates showed that the above results were not robust. Although we observed a magnitude effect (i.e., a main effect of principal amount) for both exponential (*F*(1,195) = 82.25, *p* < .001) and hyperbolic (*F*(1,195) = 71.75, *p* < .001) discount rates, the interaction effects were not statistically significant, *F*(1,195) = 3.04, *p* = .083 (exponential) *F*(1,195) = 0.46, *p* = .499 (hyperbolic). The medians and interquartile ranges for the discount rates are presented in Table S1. Therefore, the non-parametric ANOVAs did not show support for Hypothesis 1. Figure S1 visually represents the results for the exponential and hyperbolic discount rates, with boxplots of the medians and interquartile ranges. (All figures are produced using code from Allen et al., 2019.)

Table S1

*The means (and standard deviations) for the log-transformed discount rates and the medians (and interquartile ranges) for the untransformed discount rates in* ***Study 1, Gain Domain****.*

|  |  |  | **Principal Amount** | |  |
| --- | --- | --- | --- | --- | --- |
|  |  |  | **Small (£15)** | **Large (£1,500)** | **Total** |
| **log(a)** | **Frame** | **Currency (*n* = 94)** | 3.88 (1.35) | 2.82 (1.65) | 3.35 (1.59) |
|  |  | **Percent (*n* = 86)** | 3.76 (1.30) | 3.17 (1.40) | 3.46 (1.38) |
|  |  | **Total** | 3.82 (1.32) | 2.99 (1.54) |  |
| **log(r)** | **Frame** | **Currency (*n* = 94)** | 0.36 (1.15) | -0.56 (1.51) | -0.10 (1.42) |
|  |  | **Percent (*n* = 86)** | 0.27 (1.09) | -0.24 (1.22) | 0.02 (1.18) |
|  |  | **Total** | 0.32 (1.12) | -0.40 (1.39) |  |
| **log(h)** | **Frame** | **Currency (*n* = 94)** | 0.66 (1.35) | -0.40 (1.65) | 0.13 (1.59) |
|  |  | **Percent (*n* = 86)** | 0.54 (1.30) | -0.05 (1.40) | 0.25 (1.38) |
|  |  | **Total** | 0.60 (1.32) | -0.23 (1.54) |  |
| **r** | **Frame** | **Currency (*n* = 99)** | 1.15 (2.04) | 0.73 (0.89) | 1.15 (2.36) |
|  |  | **Percent (*n* = 98)** | 1.62 (2.39) | 0.89 (1.43) | 1.05 (2.41) |
|  |  | **Total** | 1.62 (2.04) | 0.73 (1.36) |  |
| **h** | **Frame** | **Currency (*n* = 99)** | 1.33 (3.20) | 0.80 (1.07) | 1.33 (3.57) |
|  |  | **Percent (*n* = 98)** | 2.00 (3.60) | 1.00 (1.80) | 1.20 (3.63) |
|  |  | **Total** | 2.00 (3.20) | 0.80 (1.73) |  |

*Note*. log(a) = log-transformed percentage premium. log(r) = log-transformed exponential discount rate. log(h) = log-transformed hyperbolic discount rate. r = exponential discount rate. h = hyperbolic discount rate. The descriptive statistics for the log-transformed discount rates are the means (and standard deviations) whereas for the untransformed discount rates they are the medians (and interquartile ranges).

*
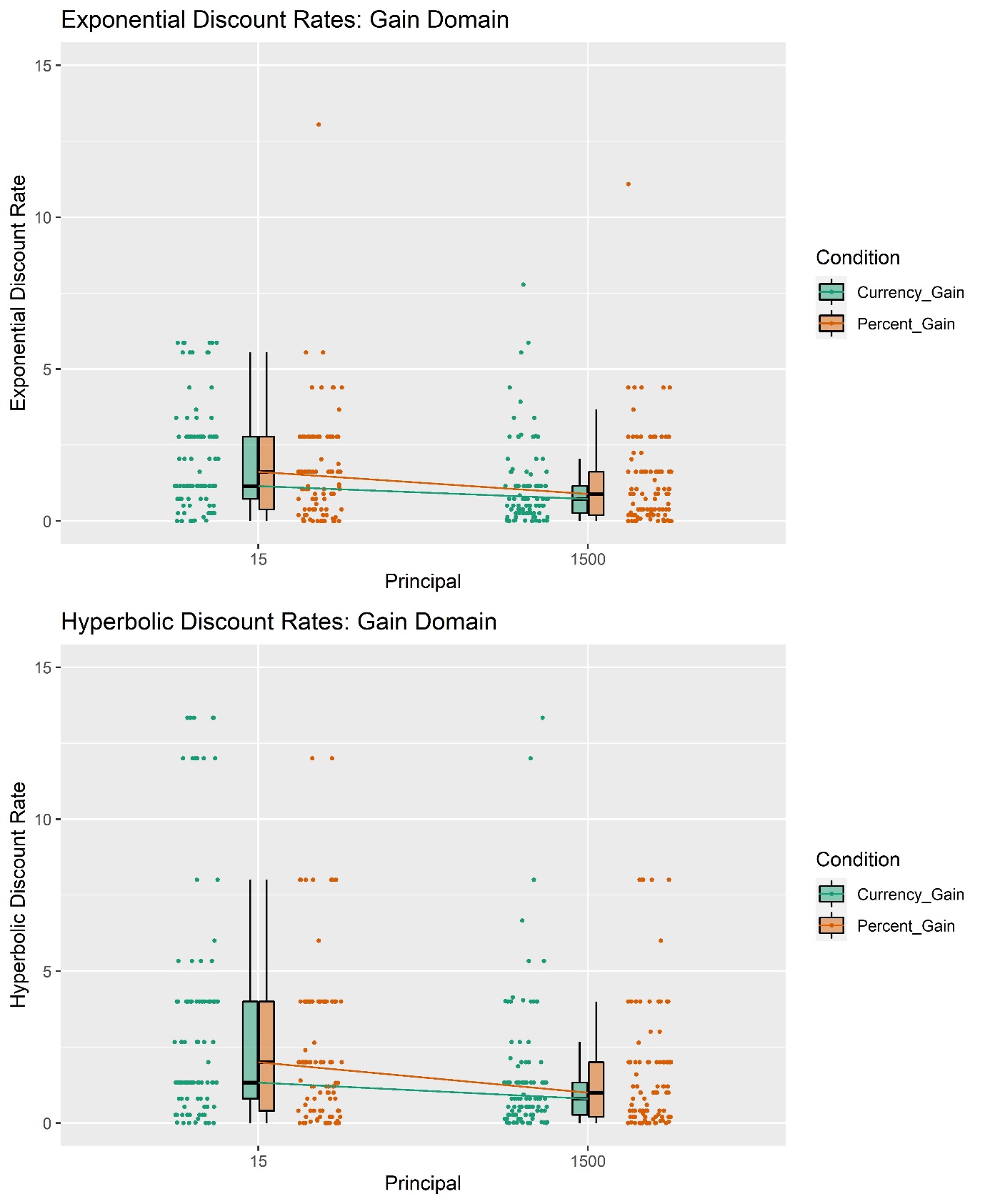
*

*Figure S1*. Study 1, Gain Domain. Median boxplot with dots representing each observation. The coloured lines connect the discount rate medians from the small to large principal amounts. Top panel: exponential discount rate. Bottom panel: hyperbolic discount rate. For the hyperbolic discount rate, 3 observations fall above the y-axis upper limit: 1 from the small principal percent frame, 1 from the large principal percent frame, and 1 from the large principal currency frame.

Taken together, we find partial, though inconclusive, support for the heuristics explanation of the magnitude effect. Replicating the well-known magnitude effect, people’s discount rates were larger for the small principal amount than for the large principal amount, and this magnitude effect was attenuated when the elicitation procedure was presented in a percent frame rather than a currency frame, but only when we analysed the log-transformed discount rates with parametric ANOVAs, not when we analysed the discount rates using non-parametric ANOVAs.

### Loss Domain

We ran the same analyses for the loss domain. In log-transforming the discount rates, we lost data from 67 participants for the parametric ANOVAs. Of these 67 participants, in the currency frame, 19 asked for 0 percentage premium for both small and large principal amounts, and 9 asked for 0 only for the small principal (nobody asked for 0 only for the large principal); and in the percent frame, 35 asked for 0 in response to both principal amounts, 2 asked for 0 only for the small principal, and 2 only for the large principal. Results for the loss domain were more consistent than for the gain domain and supported the hypothesis.

First, we report results for the log-transformed percentage premiums. The parametric ANOVA showed that there was a magnitude effect, such that (the log-transformed) percentage premiums for the small principal amount were greater than for the large principal amount, *F*(1,137) = 65.57, *p* < .001. The descriptive statistics are presented in Table 1. Importantly, the magnitude effect was attenuated by the percent frame, as indicated by the statistically significant interaction, *F*(1,137) = 23.96, *p* < .001. The magnitude effect—namely, the lower percentage premiums for the large principal compared to the small principal—was greater for participants in the currency frame, *t*(74) = 9.39, *p* < .001, *d* = 1.23, *CI_95%_*[0.89, 1.57], than for participants in the percent frame, *t*(63) = 2.23, *p* = .029, *d* = 0.24, *CI_95%_*[0.02, 0.46].

Second, we report the results for the exponential and hyperbolic discount rates. The parametric ANOVAs showed that discount rates for the small principal amount were greater than for the large principal amount, *F*(1,137) = 71.88, *p* < .001 (log-transformed exponential) *F*(1,137) = 65.57, *p* < .001 (log-transformed hyperbolic). The descriptive statistics are presented in Table S2. Importantly, the magnitude effect was attenuated by the percent frame, as indicated by the statistically significant interactions, *F*(1,137) = 27.17, *p* < .001 (log-transformed exponential) *F*(1,137) = 23.96, *p* < .001 (log-transformed hyperbolic). For (log-transformed) exponential discount rates, the magnitude effect was greater for participants in the currency frame, *t*(74) = 10.11, *p* < .001, *d* = 1.28, *CI_95%_*[0.94, 1.61], than for participants in the percent frame, *t*(63) = 2.22, *p* = .0302, *d* = 0.24, *CI_95%_*[0.02, 0.45]. Likewise for (log-transformed) hyperbolic discount rates: currency frame, *t*(74) = 9.39, *p* < .001, *d* = 1.23, *CI_95%_*[0.89, 1.57]; percent frame, *t*(63) = 2.23, *p* = .0291, *d* = 0.24, *CI_95%_*[0.02, 0.46].

The nonparametric ANOVAs, using the untransformed discount rates, also showed statistically significant magnitude effects that were attenuated by the percent frame. The medians and interquartile ranges are presented in Table S2. The main effect of principal amount was statistically significant such that discount rates were greater for the small principal than the large principal, *F*(1,204) = 76.27, *p* < .001 (exponential) *F*(1,204) = 103.67, *p* < .001 (hyperbolic). These main effects were qualified by statistically significant interactions between principal and frame, *F*(1,204) = 31.19, *p* < .001 (exponential), *F*(1,204) = 42.20, *p* < .001 (hyperbolic). Using the Wilcoxon Signed-Rank Test with continuity correction, we see that, for exponential discount rates, the magnitude effect was greater for participants in the currency frame, *Z* = -5.82, *p* < .001, *r* = 0.41, than for participants in the percent frame, *Z* = 2.63, *p* = .0085, *r* = 0.18. Likewise for hyperbolic discount rates: currency frame, *Z* = 5.86, *p* < .001, *r* = 0.41; percent frame, *Z* = 2.64, *p* = .0084, *r* = 0.18. Figure S2 visually represents the results for exponential and hyperbolic discount rates in the loss domain.

Table S2

*The means (and standard deviations) for the log-transformed discount rates and the medians (and interquartile ranges) for the untransformed discount rates in* ***Study 1,*** ***Loss Domain****.*

|  |  |  | **Principal Amount** | |  |
| --- | --- | --- | --- | --- | --- |
|  |  |  | **Small (£15)** | **Large (£1,500)** | **Total** |
| **log(a)** | **Frame** | **Currency (*n* = 75)** | 3.37 (1.09) | 1.87 (1.32) | 2.62 (1.42) |
|  |  | **Percent (*n* = 64)** | 2.64 (1.60) | 2.27 (1.47) | 2.45 (1.54) |
|  |  | **Total** | 3.03 (1.39) | 2.06 (1.40) |  |
| **log(r)** | **Frame** | **Currency (*n* = 75)** | -0.04 (0.91) | -1.42 (1.21) | -0.73 (1.27) |
|  |  | **Percent (*n* = 64)** | -0.73 (1.45) | -1.06 (1.34) | -0.89 (1.40) |
|  |  | **Total** | -0.35 (1.23) | -1.25 (1.28) |  |
| **log(h)** | **Frame** | **Currency (*n* = 75)** | 0.15 (1.09) | -1.35 (1.32) | -0.60 (1.42) |
|  |  | **Percent (*n* = 64)** | -0.58 (1.60) | -0.95 (1.47) | -0.77 (1.54) |
|  |  | **Total** | -0.19 (1.39) | -1.16 (1.40) |  |
| **r** | **Frame** | **Currency (*n* = 103)** | 0.73 (1.15) | 0.13 (0.21) | 0.26 (1.11) |
|  |  | **Percent (*n* = 103)** | 0.20 (0.89) | 0.12 (0.38) | 0.17 (0.69) |
|  |  | **Total** | 0.38 (1.15) | 0.13 (0.38) |  |
| **h** | **Frame** | **Currency (*n* = 103)** | 0.80 (1.33) | 0.13 (0.21) | 0.27 (1.29) |
|  |  | **Percent (*n* = 103)** | 0.20 (1.00) | 0.12 (0.40) | 0.18 (0.75) |
|  |  | **Total** | 0.40 (1.33) | 0.13 (0.40) |  |

*Note*. log(a) = log-transformed percentage premium. log(r) = log-transformed exponential discount rate. log(h) = log-transformed hyperbolic discount rate. r = exponential discount rate. h = hyperbolic discount rate. The descriptive statistics for the log-transformed discount rates are the means (and standard deviations) whereas for the untransformed discount rates they are the medians (and interquartile ranges).


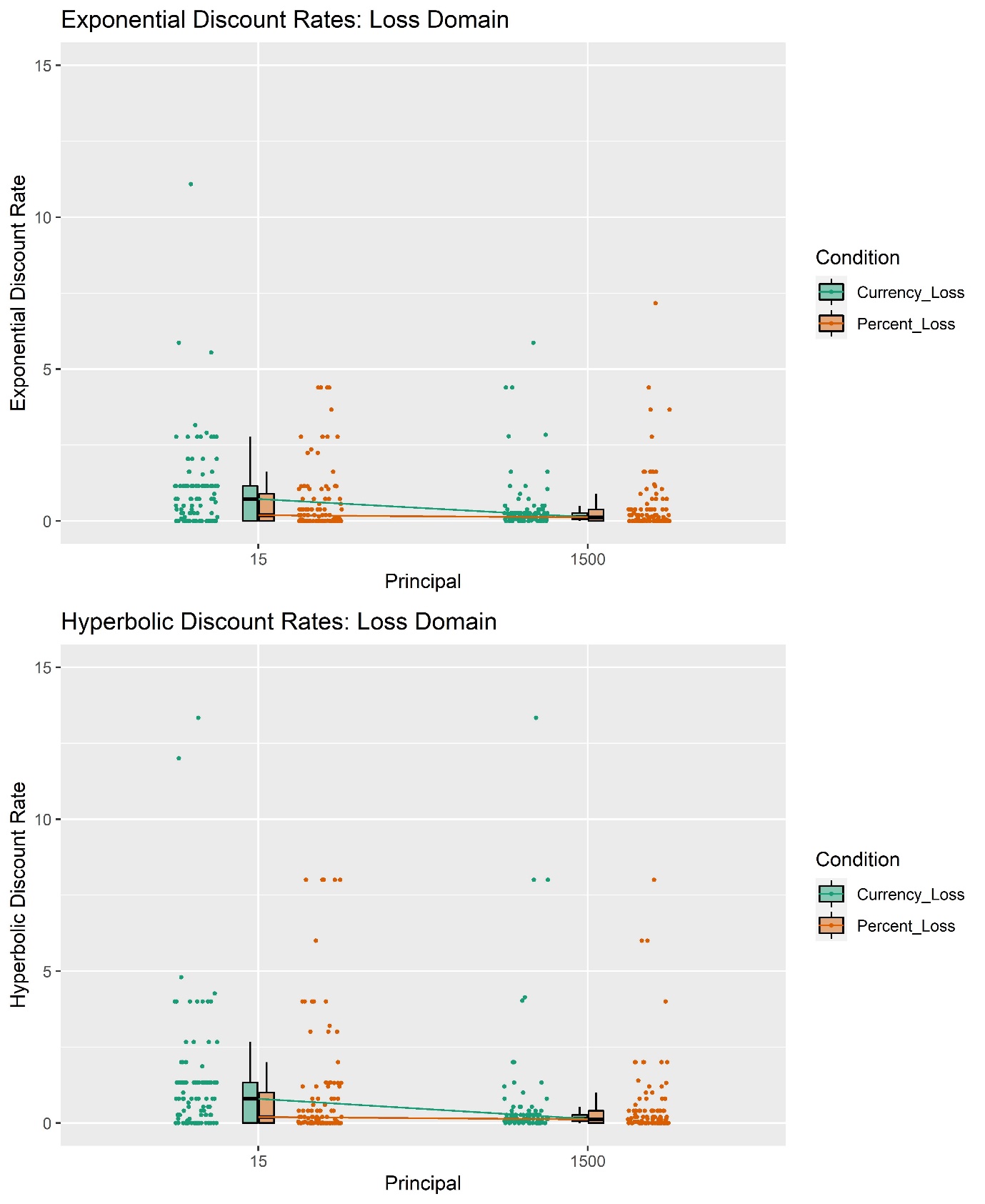


*Figure S2*. Study 1, Loss Domain. Median boxplot with dots representing each observation. The coloured lines connect the discount rate medians from the small to large principal amounts. Top panel: exponential discount rate. Bottom panel: hyperbolic discount rate. For the hyperbolic discount rate, 2 observations fall above the y-axis upper limit: 1 from the large principal percent frame, and 1 from the small principal currency frame.

In sum, results for both the parametric and non-parametric ANOVAs on both the exponential and hyperbolic discount rates converged to show that there was a magnitude effect in the loss domain and that it was attenuated by the percent frame; the hypothesis was supported.

### Parametric Analyses on Log-Transformed Variables without Data Loss

The parametric analyses for Study 1 resulted in considerable data loss due to the log-transformation of the outcome variables producing undefined values. Therefore, we also considered Judgement and Decision Making’s statistical recommendation of adding 1 (i.e., +1) to outcome variables prior to log transforming them to avoid log transforming values of zero. In this way, we can conduct the parametric analyses without any data loss. Table S3 presents the descriptive statistics for all three outcome variables, for the gain and loss domains. (a is the premium amount participants asked for in percentage points relative to the principal, r is the exponential discount rate, and h is the hyperbolic discount rate.)

#### Gain Domain

For the log-transformed percentage premiums in the gain domain, there was a nonsignificant main effect of framing, *F*(1,195) = 0.54, *p* = .462; a significant main effect of principal amount, *F*(1,195) = 61.05, *p* < .0001, reflecting a magnitude effect (see Table S3, comparing total rows of small and large principal columns); and a nonsignificant interaction, *F*(1,195) = 3.68, *p* = .0566. The pairwise comparisons shows that there were statistically significant magnitude effects in the currency frame, *t*(98) = 8.37, *p* < .0001, *d* = 0.60, *CI_95%_*[0.44, 0.75], and the percent frame, *t*(98) = 3.61, *p* = .0005, *d* = 0.33, *CI_95%_*[0.14, 0.51].

For the log-transformed exponential discount rate in the gain domain, there was a nonsignificant main effect of framing, *F*(1,195) = 0.30, *p* = .588; a significant main effect of principal amount, *F*(1,195) = 62.06, *p* < .0001, reflecting a magnitude effect; and a nonsignificant interaction, *F*(1,195) = 3.82, *p* = .0522. The pairwise comparisons shows that there were statistically significant magnitude effects in the currency frame, *t*(98) = 8.34, *p* < .0001, *d* = 0.66, *CI_95%_*[0.49, 0.83], and the percent frame, *t*(97) = 3.66, *p* = .0004, *d* = 0.39, *CI_95%_*[0.17, 0.60].

For log-transformed hyperbolic discount rate in the gain domain, there was a nonsignificant main effect of framing, *F*(1,195) = 0.26, *p* = .611; a significant main effect of principal amount, *F*(1,195) = 50.23, *p* < .0001, reflecting a magnitude effect; and a nonsignificant interaction, *F*(1,195) = 3.29, *p* = .0715. The pairwise comparisons shows that there were statistically significant magnitude effects in the currency frame, *t*(98) = 7.83, *p* < .0001, *d* = 0.64, *CI_95%_*[0.47, 0.82], and the percent frame, *t*(97) = 3.20, *p* = .0019, *d* = 0.37, *CI_95%_*[0.13, 0.60].

#### Loss Domain

For the log-transformed percentage premium in the loss domain, there was a nonsignificant main effect of framing, *F*(1,204) = 3.44, *p* = .0652; a significant main effect of principal amount, *F*(1,204) = 41.33, *p* < .0001, reflecting a magnitude effect; and a significant interaction, *F*(1,204) = 11.65, *p* = .0008. The pairwise comparisons shows that there was a statistically significant magnitude effect in the currency frame, *t*(102) = 6.28, *p* < .0001, *d* = 0.53, *CI_95%_*[0.35, 0.70], that was smaller than the magnitude effect in the percent frame, *t*(102) = 2.43, *p* = .0169, *d* = 0.15, *CI_95%_*[0.03, 0.28].

For the log-transformed exponential discount rate in the loss domain, there was a nonsignificant main effect of framing, *F*(1,204) = 1.66, *p* = .199; a significant main effect of principal amount, *F*(1,204) = 44.57, *p* < .0001, reflecting a magnitude effect; and a significant interaction, *F*(1,204) = 11.35, *p* = .0009. The pairwise comparisons shows that there was a statistically significant magnitude effect in the currency frame, *t*(102) = 6.40, *p* < .0001, *d* = 0.67, *CI_95%_*[0.44, 0.90], that was smaller than the magnitude effect in the percent frame, *t*(102) = 2.67, *p* = .0089, *d* = 0.21, *CI_95%_*[0.05, 0.37].

For the log-transformed hyperbolic discount rate in the loss domain, there was a nonsignificant main effect of framing, *F*(1,204) = 1.41, *p* = .236; a significant main effect of principal amount, *F*(1,204) = 34.33, *p* < .0001, reflecting a magnitude effect; and a significant interaction, *F*(1,204) = 8.49, *p* = .0040. The pairwise comparisons shows that the statistically significant magnitude effect in the currency frame, *t*(102) = 5.51, *p* < .0001, *d* = 0.62, *CI_95%_*[0.38, 0.86], was larger than the magnitude effect in the percent frame, *t*(102) = 2.43, *p* = .0167, *d* = 0.21, *CI_95%_*[0.04, 0.38].

Table S3

*The means (and standard deviations) for the log-transformed discount rates* ***after adding 1*** *to the discount rate, in* ***Study 1****.*

|  |  |  | **Principal Amount** | |  |
| --- | --- | --- | --- | --- | --- |
| **GAIN** | | | **Small (£15)** | **Large (£1,500)** | **Total** |
| **log(a)** | **Frame** | **Currency (*n* = 99)** | 3.74 (1.47) | 2.88 (1.45) | 3.31 (1.52) |
|  |  | **Percent (*n* = 98)** | 3.43 (1.63) | 2.90 (1.59) | 3.16 (1.63) |
|  |  | **Total** | 3.59 (1.55) | 2.89 (1.52) |  |
| **log(r)** | **Frame** | **Currency (*n* = 99)** | 0.98 (0.56) | 0.62 (0.50) | 0.80 (0.56) |
|  |  | **Percent (*n* = 98)** | 0.87 (0.57) | 0.66 (0.53) | 0.76 (0.56) |
|  |  | **Total** | 0.92 (0.56) | 0.64 (0.51) |  |
| **log(h)** | **Frame** | **Currency (*n* = 99)** | 1.19 (0.77) | 0.73 (0.65) | 0.96 (0.75) |
|  |  | **Percent (*n* = 98)** | 1.05 (0.78) | 0.77 (0.71) | 0.91 (0.76) |
|  |  | **Total** | 1.12 (0.78) | 0.75 (0.68) |  |
| **LOSS** | | |  |  |  |
| **log(a)** | **Frame** | **Currency (*n* = 103)** | 2.50 (1.77) | 1.65 (1.27) | 2.07 (1.59) |
|  |  | **Percent (*n* = 103)** | 1.82 (1.76) | 1.56 (1.55) | 1.69 (1.66) |
|  |  | **Total** | 2.16 (1.79) | 1.61 (1.41) |  |
| **log(r)** | **Frame** | **Currency (*n* = 103)** | 0.56 (0.51) | 0.25 (0.37) | 0.41 (0.47) |
|  |  | **Percent (*n* = 103)** | 0.39 (0.50) | 0.29 (0.42) | 0.34 (0.46) |
|  |  | **Total** | 0.47 (0.51) | 0.27 (0.39) |  |
| **log(h)** | **Frame** | **Currency (*n* = 103)** | 0.65 (0.68) | 0.29 (0.47) | 0.47 (0.61) |
|  |  | **Percent (*n* = 103)** | 0.45 (0.63) | 0.33 (0.53) | 0.39 (0.58) |
|  |  | **Total** | 0.55 (0.66) | 0.31 (0.50) |  |

*Note*. log(a) = log-transformed premium participants asked for in percentage points relative to principal amount. log(r) = log-transformed exponential discount rate. log(h) = log-transformed hyperbolic discount rate. Prior to log-transforming the outcome variables, we added 1 (i.e., +1) to each observation to avoid undefined values resulting in missing data for the parametric analyses.

# Study 2 – Procedure & Pre-Registered Dependent Variables

## Procedure

Participants were instructed that they would first complete a practice task asking them about someone else’s preferences that they had to answer correctly before moving on to the next task which would ask them about their own preferences. The preliminary task asked participants (italics are for participants in the currency frame and the square brackets for participants in the percent frame):

Imagine that someone is owed £100 and they said that they'd be just as happy to receive the £100 immediately or postpone and receive a bonus of *£50* [50%] on top of the £100 later.

Based on this, the person feels the same about receiving £100 now and receiving £100 plus a bonus of *£* __ [%] later.

The second preliminary question was worded in exactly the same way but the £50 (or 50%) bonus was replaced with a £200 (or 200%) bonus. After answering both preliminary questions correctly, all participants were given the following instructions:

Imagine that there was a legitimate error on your back taxes in your favor (that is, you paid more taxes than you had to), and that you are given two options for receiving your credit.

**Important!** You can have your money transferred immediately in your back account, or in 3 months from now with the addition of a bonus.

Following these instructions participants answered two questions (presented in random order) by filling in the blanks (italics for participants in the currency frame and square brackets for participants in the percent frame):

SCENARIO 1. You are owed £15.

How much of a bonus (in *£* [%]) would make receiving the **£15 + bonus** in 3 months just the same as receiving the £15 now?

I feel the same about receiving £15 now and receiving **£15 + *£*__[%] in 3 months.**

The second scenario was exactly the same except that the small principal amount of £15 was replaced with a larger principal amount of £1,500. After this task, we asked participants their sex, year of birth, education level, to rate the clarity of the questions in the survey (from 1 = *not clear at all*, to 7 = *very clear*; *M* = 5.51, *SD* = 1.70), and, optionally, to provide comments on the clarity of the questions.

## Measures

The outcome variables, the exponential and hyperbolic discount rates, were calculated in exactly the same way as in Study 1. Because we expected both discount rates to be highly skewed, we pre-registered our intention to test the hypothesis using non-parametric ANOVAs with Aligned Rank Transform on the untransformed discount rates. Like in Study 1, we applied the Holm-Bonferroni correction for multiple comparisons: The interaction with the lowest *p*-value was tested against an alpha of .025 and the other against an alpha of .05. We did not intend, nor did we use the log-transformed discount rates.

## Results

To the hypothesis that the magnitude effect would be attenuated by the percent frame, we used 2 non-parametric ANOVAs, with principal amount as the within-subjects factor and framing as the between-subjects factor, using the exponential discount rate and hyperbolic discount rate as the dependent variables. Table S4 presents the descriptive statistics. We observed an overall magnitude effect for both discount rates. That is, the discount rate was greater for small principals than for large principals (see Table S4), as indicated by statistically significant main effects of principal amount: *F*(1,259) = 132.93, *p* < .001 (exponential discount rate) and *F*(1,259) = 131.66, *p* < .001 (hyperbolic discount rate). Critically, both of these main effects were qualified by statistically significant interactions with the framing factor: *F*(1,259) = 4.05, *p* = .0454 (exponential discount rate) and *F*(1,259) = 11.46, *p* = .0008 (hyperbolic discount rate). Using the Wilcoxon Signed-Rank Test with continuity correction to probe into the interaction further, we found that, for exponential discount rates, the magnitude effect was greater for participants in the currency frame, *Z* = 7.07, *p* < .001, *r* = 0.49, than for participants in the percent frame, *Z* = 5.73, *p* < .001, *r* = 0.40. Likewise for hyperbolic discount rates: currency frame, *Z* = 6.97, *p* < .001, *r* = 0.49; percent frame, *Z* = 5.72, *p* < .001, *r* = 0.40. Figure S3 visually represents the results for the exponential and hyperbolic discount rates. In sum, the results of both the exponential and hyperbolic discount rates showed support for the hypothesis: The magnitude effect was attenuated by the percent frame.

Table S4

*The medians (and interquartile ranges) for the untransformed discount rates in* ***Study 2,*** ***Gain Domain****.*

|  |  |  | **Principal Amount** | |  |
| --- | --- | --- | --- | --- | --- |
|  |  |  | **Small (£15)** | **Large (£1,500)** | **Total** |
| **r** | **Frame** | **Currency (*n* = 129)** | 2.04 (2.24) | 1.15 (1.03) | 1.15 (2.04) |
|  |  | **Percent (*n* = 132)** | 1.62 (2.06) | 0.89 (1.24) | 1.35 (1.86) |
|  |  | **Total** | 1.62 (1.62) | 1.05 (1.12) |  |
| **h** | **Frame** | **Currency (*n* = 129)** | 2.67 (4.00) | 1.33 (1.33) | 1.33 (3.20) |
|  |  | **Percent (*n* = 132)** | 2.00 (3.22) | 1.00 (1.60) | 1.60 (2.60) |
|  |  | **Total** | 2.00 (2.67) | 1.20 (1.47) |  |

*Note*. r = exponential discount rate. h = hyperbolic discount rate.


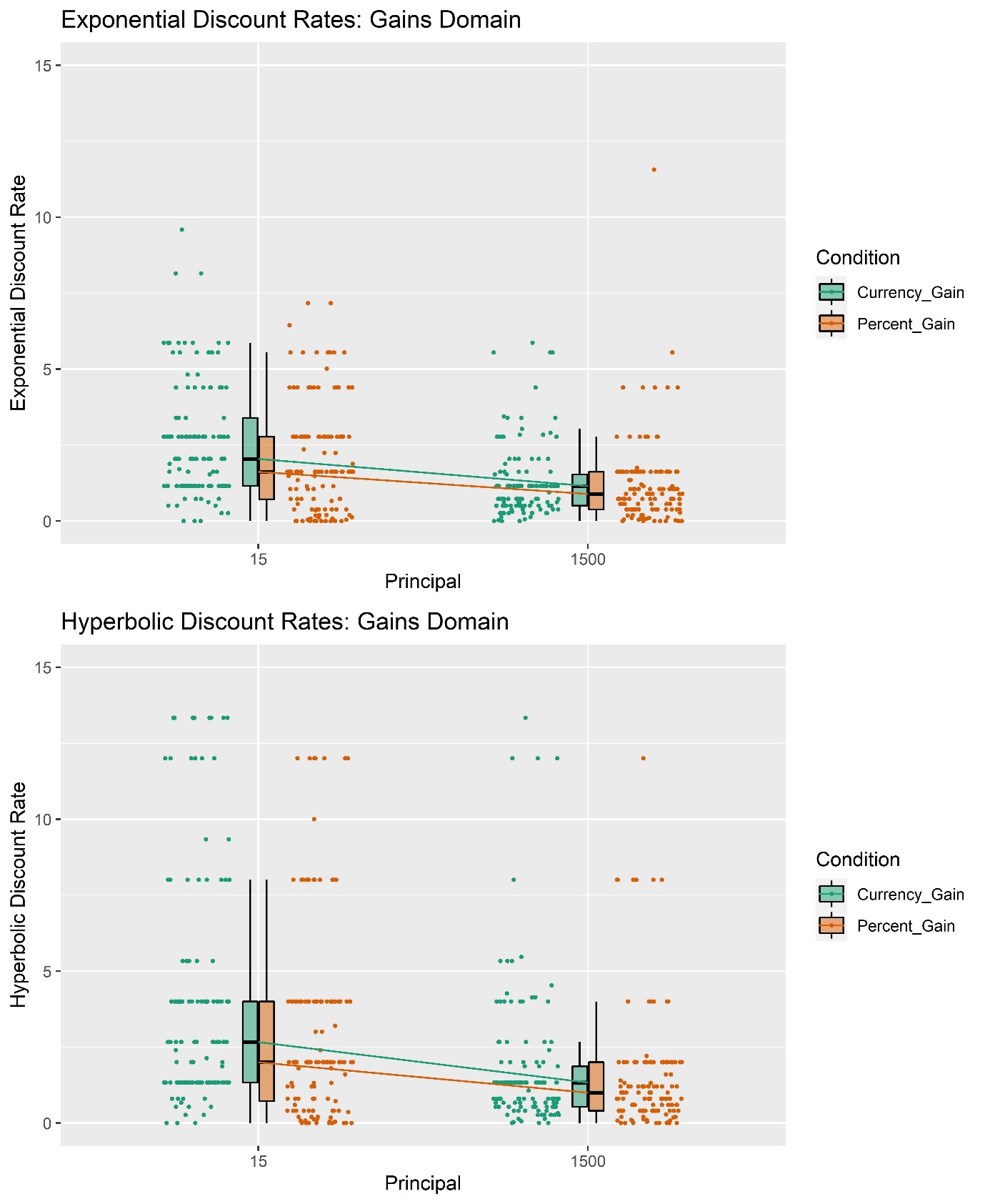


*Figure S3*. Study 2, Gain Domain. Median boxplot with dots representing each observation. The coloured lines connect the discount rate medians from the small to large principal amounts. Top panel: exponential discount rate. Bottom panel: hyperbolic discount rate. For the hyperbolic discount rate, 7 observations fall above the y-axis upper limit: 1 from the large principal percent frame, 3 from the small principal percent frame, and 3 from the small principal currency frame. We note that even after stepwise removal of outliers falling outside of the limits of the y-axis in the above figure, our conclusions and inferential results were qualitatively unchanged.

# Study 3

## Procedure

Similar to Study 1 and Study 2, participants were instructed that they would first need to do a practice task comprising of two preliminary questions. The preliminary questions were phrased exactly the same as in Study 1 and Study 2, with the same framing and domain as the condition to which participants were randomly assigned to. After answering both preliminary questions correctly, participants were asked to answer the following questions (presented in random order and on separate pages) by filling in the blanks (italics for gain domain and square brackets for losses):

SCENARIO 1

Imagine that there was a legitimate error on your back taxes in *your* [the government's] favor (that is, you paid *more* [less] taxes than you had to), and that you are given two options for *receiving* [paying] your *credit* [debt]. You can have your money *transferred* [withdrawn] *in* [from] your bank account immediately, or in **3 months** from now with the addition of a *bonus* [fee].

Imagine that *the government owes you* [you owe the government] **£45**. How much of a *bonus* [fee] (in £) would make *receiving* [paying] the **£45 + £ *bonus* [fee]** in 3 months, just the same to you as *receiving* [paying] the **£45 now**?

*Bonus* [Fee] (in £): ___

The second question (SCENARIO 2) was exactly the same except that the small principal amount of £45 was replaced with a larger principal amount of £1,300. For participants in the percent frame condition the pounds symbol (£) was replaced with the percent symbol (%) following the monetary amount.

After this task, we asked participants their sex, year of birth, and, optionally, to provide spontaneous comments on the survey.

# Study 4

## Procedure

Similar to Study 1, Study 2, and Study 3, participants were instructed that they would first need to do a practice task comprising of two preliminary questions. The preliminary questions were phrased exactly the same as in Study 1, Study 2, and Study 3, but only in the loss domain framing. After answering both preliminary questions correctly, participants were asked to answer the following questions (presented in random order and on separate pages) by filling in the blanks (italics are for participants in the currency frame and the square brackets for participants in the percent frame):

SCENARIO 1

Imagine that there was a legitimate error on your back taxes in the government's favor (that is, you paid less taxes than you had to), and that you are given two options for paying your debt. You can have your money withdrawn from your bank account immediately, or in **3 months** from now with the addition of a fee.

Imagine that you owe the government **£12**. How much of a fee (in *£* [%]) would make paying the **£12 + *£* [%] fee** in 3 months, just the same to you as paying the **£12 now**?

Fee (in *£* [%]): ___

The second question (SCENARIO 2) was exactly the same except that the small principal amount of £12 was replaced with a larger principal amount of £750.

After this task, we asked participants their sex, year of birth, and, optionally, to provide spontaneous comments on the survey.

# Additional Analyses to Rule out Alternative Explanations

In the manuscript we put forth an alternative and competing explanation for the results. We suggested that the percent frame may have attenuated the magnitude effect by allowing participants to respond consistently more easily for the two principal amounts. An anonymous reviewer suggested that this alternative explanation could potentially be addressed by analysing only participants’ first responses, comparing those who responded first to the small principal amount with those who responded first to the large principal amount. In effect, this would be examining the magnitude effect in a between-subjects comparison. Although we note that in Studies 1 and 2 participants were presented with the two principal amounts (in random order) on the same page, we can analyse the data as suggested by the reviewer under the assumption that (most or many) participants did not go back to change their responses to the question that they answered first. If the attenuation of the magnitude effect by the percent frame is due to the ease with which participants could remain consistent in their responses across the different principal amounts, then we would expect to not observe the attenuation effect when we analyze only participants’ first responses. Moreover, Studies 3 and 4 presented participants with the two principal amounts on separate pages so that participants could not go back to change their answers. As can be seen in the results reported in Tables S5 and S6, there is general support for the hypothesis that the percentage framing attenuates the magnitude effect for both gains and losses. Table S5 presents the descriptive statistics, and Table S6 presents the inferential statistics.

For the domain of gains, there was a consistent magnitude effect (Studies 1, 2, and 3) as indicated by the statistically significant main effects of principal amount: People asked for a larger relative bonus in response to the small principal than the large principal. Most importantly, and in line with the hypothesis, there was a statistically significant interaction effect between the principal amount and the framing condition. Specifically, as can be seen from the simple effects analyses and effect sizes reported below, the magnitude effect was consistently attenuated by the percent frame: The size of the magnitude effect in the currency frame was larger than the size of the magnitude effect in the percent frame.

For the domain of losses, we observed a magnitude effect in Studies 1 and 4, but in Study 3 neither the main effect of principal amount nor the interaction were statistically significant. Nonetheless, across all three studies there was a magnitude effect in the currency frame that was (statistically significantly in Studies 1 and 4, and descriptively in Study 3) attenuated in the percent frame, as can be seen in the simple effects analyses reported below.

The following simple effects analyses are the Wilcoxon Signed-Rank Test with continuity correction for the untransformed premium. For the domain of gains in Study 1, the magnitude effect in the currency frame (Study 1: *Z* = -5.65, *p* < .0001, *r* = 0.57; Study 2: *Z* = -6.77, *p* < .0001, *r* = 0.60; Study 3: *Z* = -5.83, *p* < .0001, *r* = 0.47) was larger than in the percent frame (Study 1: *Z* = -2.70, *p* = .0070, *r* = 0.27; Study 2: *Z* = -3.65, *p* = .0003, *r* = 0.32; Study 3: *Z* = -5.83, *p* = .1148, *r* = 0.13). For the domain of losses, the magnitude effect in the currency frame (Study 1: *Z* = -4.14, *p* < .0001, *r* = 0.41; Study 3: *Z* = -1.56, *p* = .1194, *r* = 0.13; Study 4: *Z* = -4.44, *p* < .0001, *r* = 0.36) was larger than in the percent frame (Study 1: *Z* = -0.21, *p* = .8325, *r* = 0.02; Study 3: *Z* = -0.60, *p* = .5462, *r* = 0.05; Study 4: *Z* = -0.98, *p* = .3295, *r* = 0.08), though the difference was not statistically significant in Study 3. These results show support for the hypothesis in the domain of gains and losses.

Table S5

*The means (and standard deviations) for the log-transformed premium, log(a), and the medians (and interquartile ranges) for the untransformed premium, a, in* ***Studies 1, 2, 3, and 4 examining only participants’ response to the principal amount they saw first****.*

|  |  |  | **Principal Amount** | |  |
| --- | --- | --- | --- | --- | --- |
| **Study 1** - **Gain** | |  | **Small** | **Large** | **Total** |
|  | **Frame** | **Currency** | 100 (100) *n=*50 | 13.33 (26.67) *n=*49 | 33.33 (88.33) |
|  |  | **Percent** | 50 (90) *n=*45 | 10 (45) *n=*53 | 25 (45) |
|  |  | **Total** | 50 (75) | 13.33 (28.33) |  |
| **Study 1 -** **Loss** | | |  |  |  |
|  | **Frame** | **Currency** | 33.33 (43.33) *n=*57 | 3.33 (5.67) *n=*46 | 6.67 (31.67) |
|  |  | **Percent** | 5 (30) *n=*45 | 4 (10) *n*=58 | 5 (20) |
|  |  | **Total** | 18.33 (33.33) | 3.33 (10.00) |  |
| **Study 2 - Gain** | |  |  |  |  |
|  | **Frame** | **Currency** | 66.67 (66.67) *n=*61 | 20 (16.67) *n=*68 | 33.33 (80) |
|  |  | **Percent** | 50 (80) *n=*66 | 20 (40) *n=*66 | 42.50 (40) |
|  |  | **Total** | 53.33 (66.67) | 20 (25) |  |
| **Study 3 - Gain** | |  |  |  |  |
|  | **Frame** | **Currency** | 55.56 (66.67) *n=*65 | 19.23 (21.15) *n=*90 | 33.33 (80) |
|  |  | **Percent** | 30 (40) *n=*70 | 20 (30) *n=*77 | 42.50 (40) |
|  |  | **Total** | 53.33 (66.67) | 20 (25) |  |
| **Study 3 - Loss** | |  |  |  |  |
|  | **Frame** | **Currency** | 6.67 (22.22) *n=*76 | 3.04 (7.69) *n=*78 | 3.85 (11.11) |
|  |  | **Percent** | 1 (10) *n=*63 | 2.75 (10) *n=*74 | 2 (10) |
|  |  | **Total** | 2.22 (11.11) | 3 (10) |  |
| **Study 4 - Loss** | |  |  |  |  |
|  |  | **Currency** | 16.67 (41.67) *n=*69 | 2.67 (6.67) *n=*81 | 6.67 (20) |
|  |  | **Percent** | 5 (20) *n=7*3 | 2.5 (10) *n=*74 | 3 (14) |
|  |  | **Total** | 10 (33.33) | 2.67 (6.67) |  |

*Note*. The descriptive statistics are the medians (and interquartile ranges).

Table S6

*The inferential statistics from 2 (principal amount: small vs large) by 2 (framing: currency vs percent) between subjects ANOVAs on the untransformed premium, a, in* ***Studies 1, 2, 3, and 4 examining only participants’ response to the principal amount they saw first****.*

| Study | Effect | Inferential Statistics |
| --- | --- | --- |
| Study 1 - Gain | Principal | *F*(1,193) = 51.30, *p* < .0001 |
|  | Framing | *F*(1,193) = 13.76, *p* = .0003 |
|  | Principal*Framing | *F*(1,193) = 10.34, *p* = .0015 |
| Study 1 - Loss | Principal | *F*(1,202) = 26.20, *p* < .0001 |
|  | Framing | *F*(1,202) = 11.58, *p* = .0008 |
|  | Principal*Framing | *F*(1,202) = 13.62, *p* = .0003 |
| Study 2 - Gain | Principal | *F*(1,257) = 37.98, *p* < .0001 |
|  | Framing | *F*(1,257) = 8.07, *p* = .0049 |
|  | Principal*Framing | *F*(1,257) = 4.24, *p* = .0405 |
| Study 3 - Gain | Principal | *F*(1,298) = 46.72, *p* < .0001 |
|  | Framing | *F*(1,298) = 9.90, *p* = .0018 |
|  | Principal*Framing | *F*(1,298) = 22.86, *p* < .0001 |
| Study 3 - Loss | Principal | *F*(1,287) = 2.53, *p* = 1126 |
|  | Framing | *F*(1,287) = 0.61, *p* = .4366 |
|  | Principal*Framing | *F*(1, 287) = 1.20, *p* = .2738 |
| Study 4 - Loss | Principal | *F*(1,293) = 36.39, *p* < .0001 |
|  | Framing | *F*(1,293) = 11.04, *p* = .0010 |
|  | Principal*Framing | *F*(1,293) = 22.17, *p* < .0001 |

*Note*. We used the nonparametric ANOVA as described in the manuscript.

**References (that are not provided in the reference list of the main text)**

Mazur, J. E. (1987). *An adjusting procedure for studying delayed reinforcement.* In M. L. Commons, J. E. Mazur, J. A. Nevin, & H. Rachlin (Eds.), *Quantitative analyses of behavior, Vol. 5. The effect of delay and of intervening events on reinforcement value* (p. 55–73). Lawrence Erlbaum Associates, Inc.

Laibson, D. (1997). Golden eggs and hyperbolic discounting. *The Quarterly Journal of Economics, 112*, 443–478. https://doi.org/10.1162/003355397555253

Strotz, R. H. (1955-1956). Myopia and inconsistency in dynamic utility maximization. *Review of Economic Studies*, 23(3), 165-180. DOI: 10.2307/2295722 https://www.jstor.org/stable/2295722

1. The quasi-hyperbolic discount model (Mazur, 1987) includes one free parameter. [↑](#footnote-ref-1)
