# Supplementary material for: Percent framing attenuates the magnitude effect in a preference-matching task of intertemporal choice
Source: PLoS One. 2022 Jan 24;17(1):e0262620. doi: 10.1371/journal.pone.0262620 (PMC8786190; doi:10.1371/journal.pone.0262620)
Supplement: S2 File — Can also be found here https://osf.io/qgxpf/. (DOC) [file pone.0262620.s004.doc]

# Supplementary Materials 2

The methods, sampling procedure, and analysis plan for Studies 1, 2, and 3 were pre-registered on the Open Science Framework (<https://osf.io/48tza>, <https://osf.io/9f4ws>, [https://osf.io/46ycf](https://osf.io/https:/osf.io/46ycf) respectively).[[1]](#footnote-2) However, following the review procedure, we had to adjust our analyses for two reasons. First, the data were highly skewed and there were unequal variances between the groups. Second, the nonparametric ANOVAs and the nonparametric analysis of simple effects that we pre-registered and originally conducted targeted different statistical variables. The nonparametric ANOVAs were on the adjusted means, whereas the nonparametric simple effects were on the medians. An anonymous reviewer suggested an alternative way to conduct the analyses. In this paper, therefore, we report improved analyses that weren’t pre-registered, but which better address the problems inherent to the nature of our data and harmonize the assessment of the overall and simple effects. We report the pre-registered analyses in full in the Supplemental Materials 1 and Supplemental Materials 2 (see here <https://osf.io/qgxpf/>). The conclusions remain qualitatively unchanged.

# Study 1

## Method

### Participants

Read et al. (2013) observed that the magnitude effect was moderated by framing with an effect size of partial η^2^ of 0.08. We took a conservative approach and powered our study to detect an effect size of partial η^2^ of 0.06. Correcting for two dependent variables (see Measures below), our Holm-Bonferroni corrected alpha for the lowest p-value that we observe for the main hypothesis, with respect to the interaction effect, will be .025. Using G*Power (Faul et al., 2007), with options set to “As in Cohen (1988),” we conducted power analyses to detect an effect size of partial η^2^ of 0.06 (Cohen’s f(V) = 0.25), alpha .025, 90% power, we required a total sample size of 202 to test the interaction hypothesis for the gain domain (i.e., 101 per condition for the between-subjects factor of frame). We similarly aimed for an additional 202 participants for the loss domain. After pre-registered exclusions, and keeping only the first entry of 1 participant for whom we had two entries, we recruited a total of 403 participants using the Prolific platform (<https://www.prolific.co/>) using prescreening criteria to include only participants with U.K. residency and who were fluent in English; 279 female, 121 male, and 3 other, mean age 36.6 years (*SD* = 11.5). Due to the randomization feature of our software there was variability in the number of participants in each condition.

### Design

In Study 1, we conducted an experiment with 8 conditions; a 2 (domain: gains vs losses) by 2 (principal amount: small vs large) by 2 (framing: currency vs percent) mixed design, with domain and framing being between-subjects factors and principal amount a within-subjects factor. Importantly, we wanted to test the attenuation hypothesis in the gains and losses domain separately. To test the attenuation hypothesis in the domain of gains, we used a 2 (principal amount: small vs large) by 2 (frame: currency vs percent) mixed design. Principal amount was a within-subjects factor and frame was a between-subjects factor. To test the attenuation hypothesis in the domain of losses, we used the same mixed design.

### Procedure

Participants first answered two preliminary questions, by typing into a text box, to become familiarized with what the task involved and to make sure they understood the questions and were paying attention to the task. The preliminary questions were presented with the same framing and domain as the condition to which participants were randomly allocated. Participants were asked to fill in the blank (italics are for the gain domain and square brackets for the loss domain):

Imagine that someone *is owed* [owes] £100 by the government and they said that they'd feel just the same about *receiving* [paying] the £100 immediately or postponing and *receiving* [paying] a *bonus* [fee] of £50 on top of the £100 later.

Based on this, we can assume that they are indifferent between *receiving* [paying] £100 now and *receiving* [paying] £100 plus a *bonus* [fee] of £ __ later.

The second preliminary question that participants had to answer correctly was worded in exactly the same way but it replaced the £50 bonus/fee with a £200 bonus/fee. For the percent frame, the pound (£) symbol was replaced with the percent symbol (%) appearing after the numerical digits. If participants answered one of these questions incorrectly, then they received a warning that they had one attempt left to give correct answers to the preliminary questions. After a second incorrect attempt, they were excluded from participating further.

After the preliminary questions, participants were given the following instructions (italics for gains and square brackets for losses):

Imagine that there was a legitimate error on your back taxes in *your* [the government’s] favor (that is, you paid *more* [less] taxes than you had to), and that you are given two options for *receiving* [paying] your *credit* [debt]. You can have your money *transferred* [withdrawn] immediately *in* [from] your bank account, or in 3 months from now with the addition of a *bonus* [fee].

In the next two scenarios, you will have to indicate HOW MUCH OF A *BONUS* [FEE] (IN BRITISH POUNDS, ON TOP OF THE ORIGINAL AMOUNT) WOULD MAKE *RECEIVING* [PAYING] THE MONEY LATER JUST THE SAME AS *RECEIVING* [PAYING] THE MONEY NOW.

Following these instructions, participants were to answer two questions (presented in random order) by filling in the blanks (italics for gain domain and square brackets for losses):

You *are owed* [owe] £15. How much of a *bonus* [fee] (in £) would make *receiving* [paying] the **£15 + *bonus* [fee]** in 3 months just the same as *receiving* [paying] the £15 now?

I am indifferent between *receiving* [paying] £15 now and *receiving* [paying] £15 plus a *bonus* [fee] of £__ in 3 months.

The second question was exactly the same except that the small principal amount of £15 was replaced with a larger principal amount of £1,500. For participants in the percent frame condition the pounds symbol (£) was replaced with the percent symbol (%) following the monetary amount.

After this task, we asked participants their sex, year of birth, education level, and, optionally, to provide spontaneous comments on the survey.

### Measures

The outcome variable is the bonus that people asked for in percentage points relative to the principal amount: We refer to this measure as the rate *a*, or the percentage premium. The formula for the percentage premium for participants in the currency frame is *a = 100 * Bonus / Principal*. The percentage premium for participants in the percent frame is simply their response which reflected the percentage of the principal that they required as a bonus for waiting.

## Results

We report results from analyses using nonparametric Aligned Rank Transform ANOVAs (from here referred to as nonparametric ANOVA) using the percentage premium as the outcome variable. A description of the Aligned Rank Transform procedure is given by Wobbrock et al. (2011), and is implemented in the ARTool package for R.

In exploratory analyses, we conducted a 3-way nonparametric ANOVA with domain, principal amount, and framing as the factors. In line with past research (Baker et al., 2003), we found that the magnitude effect was smaller in the domain of losses than in the domain of gains, as indicated by the 2-way interaction between domain and principal amount (*F*(1, 399) = 58.94, *p* < .0001). As can be seen in Table S1, the percentage premium for the small principal was larger than the percentage premium for the large principal (i.e., magnitude effect), and this magnitude effect was statistically smaller in the loss domain.

### Gain Domain

We first tested the hypothesis that the magnitude effect will be attenuated by the percentage frame in the gain domain. Although we observed a magnitude effect (i.e., a main effect of principal amount; *F*(1, 195) = 71.75, *p* < .001), the interaction effect was not statistically significant (*F*(1, 195) = 0.46, *p* = .499). The medians and interquartile ranges for the percentage premiums are presented in Table S1. Therefore, the nonparametric ANOVA did not show support for the hypothesis in the gain domain. Figure S1 visually represents the results for the untransformed percentage premiums, with boxplots of the medians and interquartile ranges. (All figures are produced using code from Allen et al., 2019.) Table S1 and Figure S1 show that the magnitude effect may be larger in the percent frame than in the currency frame, contradicting our hypothesis. However, this difference was not statistically significant (i.e., nonsignificant interaction), so we do not draw inferences about this.

Nonetheless, we report the nonparametric simple effects analyses using Wilcoxon Signed-Rank Test with continuity correction, so that additional descriptive statistics (e.g., standardized effect size estimates) can be observed. In the currency frame, we observe a magnitude effect such that the median premium for the small principal was statistically significantly greater than the median premium for the large principal (*Z* = -6.28, *p* < .0001, *r* = .45). We also observed a magnitude effect in the percent frame (*Z* = -3.03, *p* = .0025, *r* = .22). As can be seen from the standardized effect size estimates, the magnitude effect was descriptively larger in the currency (*r* = .45) than the percent frame (*r* = .22). As noted above, however, this difference is not statistically significant (i.e., nonsignificant interaction). Yet, it is worth noting that although the median differences may seem to suggest a larger magnitude effect in the percent frame, once the variability is taken into account, as is done with the standardized effect size estimates, the pattern of results are in the predicted direction. Still, we advise caution in interpreting these results, given the nonsignificant interaction effect.

Table S1

*The medians (and interquartile ranges) for the percentage premiums, a, in Studies 1, 2, 3, and 4*

|  |  |  | **Principal Amount** | |  |
| --- | --- | --- | --- | --- | --- |
| **Study 1** - **Gains** | |  | **Small (£15)** | **Large (£1,500)** | **Total** |
| **Frame** | | **Currency (*n* = 99)** | 33.33 (80.00) | 20.00 (26.67) | 33.33 (89.17) |
|  |  | **Percent (*n* = 98)** | 50.00 (90.00) | 25.00 (45.00) | 30.00 (90.63) |
|  |  | **Total** | 50.00 (80.00) | 20.00 (43.33) |  |
| **Study 1 -** **Losses** | |  | **Small (£15)** | **Large (£1,500)** |  |
| **Frame** | | **Currency (*n* = 103)** | 20.00 (33.33) | 3.33 (5.33) | 6.67 (32.33) |
|  |  | **Percent (*n* = 103)** | 5.00 (25.00) | 3.00 (10.00) | 4.45 (18.75) |
|  |  | **Total** | 10.00 (33.33) | 3.33 (10.00) |  |
| **Study 2 - Gains** | |  | **Small (£15)** | **Large (£1,500)** |  |
| **Frame** | | **Currency (*n* = 129)** | 66.67 (100.00) | 33.33 (33.33) | 33.33 (80.00) |
|  |  | **Percent (*n* = 132)** | 50.00 (80.50) | 25.00 (40.00) | 40.00 (65.00) |
|  |  | **Total** | 50.00 (66.67) | 30.00 (36.67) |  |
| **Study 3 - Gains** | |  | **Small (£45)** | **Large (£1,300)** |  |
| **Frame** | | **Currency (*n* = 155)** | 44.44 (77.78) | 23.08 (30.77) | 32.05 (52.78) |
|  |  | **Percent (*n* = 147)** | 30 (40) | 20 (40) | 25 (40) |
|  |  | **Total** | 44.44 (88.89) | 20 (30.77) |  |
| **Study 3 - Losses** | |  | **Small (£45)** | **Large (£1,300)** |  |
| **Frame** | | **Currency (*n* = 154)** | 8.33 (22.22) | 3.85 (7.67) | 3.85 (11.11) |
|  |  | **Percent (*n* = 137)** | 2 (10) | 2.5 (10) | 2 (10) |
|  |  | **Total** | 4.44 (11.11) | 3.08 (9.81) |  |
| **Study 4 - Losses** | |  | **Small (£12)** | **Large (£750)** |  |
| **Frame** | | **Currency (*n* = 151)** | 8.33 (41.67) | 3.33 (6.67) | 4.17 (22.5) |
|  |  | **Percent (*n* = 147)** | 2 (20) | 5 (10) | 3 (12) |
|  |  | **Total** | 5 (25) | 3.33 (10) |  |

*Note*. Each cell contains the bonus amount, as a percentage of the principal amount, that participants required on top of the principal amount to make the future amount equivalent to the present amount. The descriptive statistics are the medians (and interquartile ranges).

*
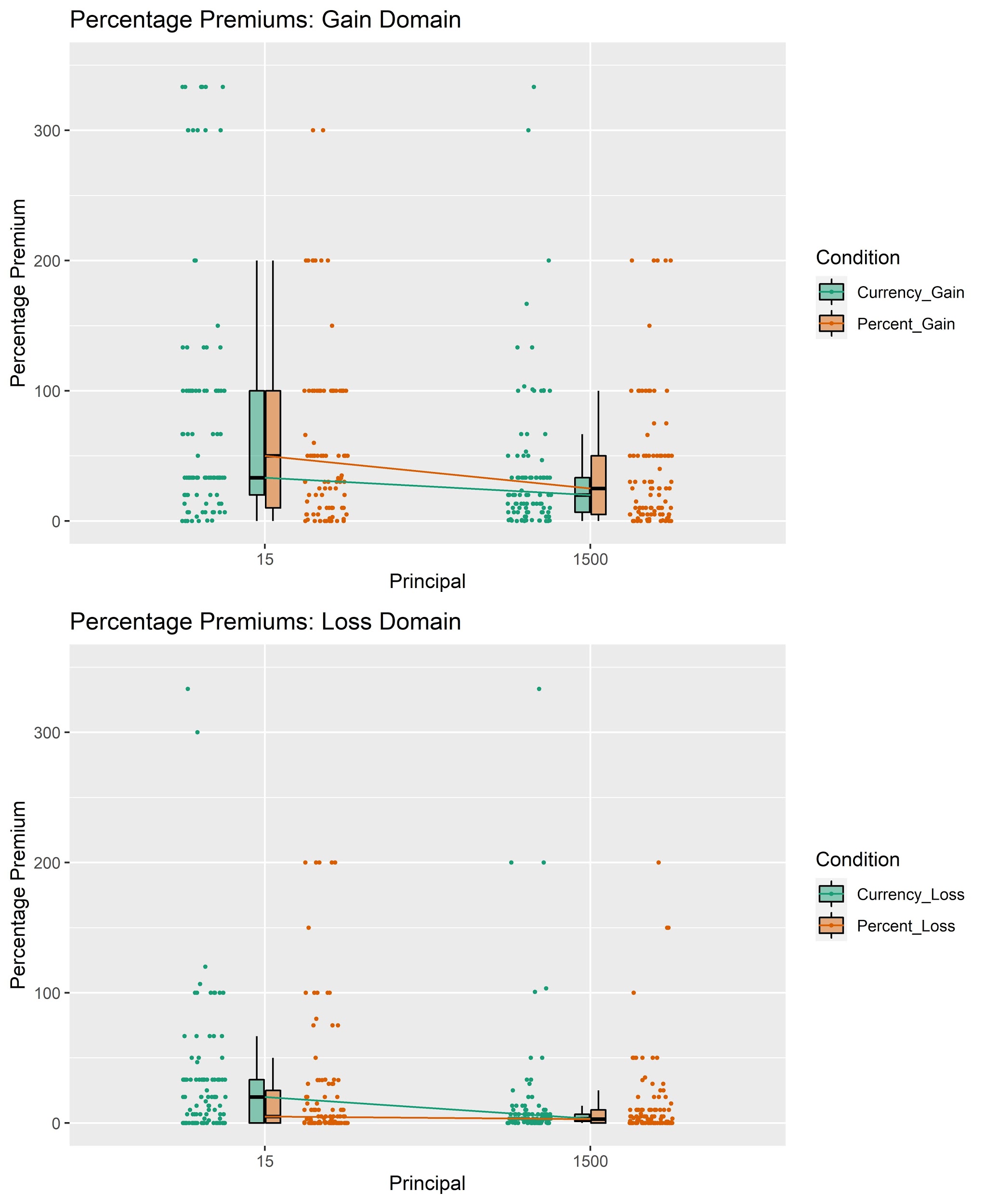
*

*Figure S1*. Study 1. The upper panel displays the results for gains and the lower panel for losses. Median boxplot with each observation represented by a dot. The lower and upper hinges of the box represent the 25th and 75th percentiles, respectively, the distance between them being the interquartile range (IQR). The upper whisker extends to the highest percentage premium that is no further from the upper hinge than 1.5*IQR, and the lower whisker extends to the lowest percentage premium that is no further than 1.5*IQR from the lower hinge. The line inside each box represents the median of the percentage premium. The coloured lines connect the medians of the percentage premium from the small to large principal amounts. A downward slope suggests a magnitude effect; the steeper the slope the larger the magnitude effect. Note that for gains 3 observations fall above the y-axis upper limit: 1 from the small principal percent frame, 1 from the large principal percent frame, and 1 from the large principal currency frame. For losses, 2 observations fall above the y-axis upper limit: 1 from the large principal percent frame, and 1 from the small principal currency frame.

Taken together, although we did not find support for the hypothesis in the gain domain, the results are inconclusive. Replicating the well-known magnitude effect, people’s percentage premiums were larger for the small principal amount than for the large principal amount. However, this magnitude effect was not attenuated by the percent frame (i.e., a nonsignificant interaction effect), although the standardized effect size estimate was descriptively smaller in the percent frame than in the currency frame.

### Loss Domain

Results for the loss domain supported the hypothesis. The nonparametric ANOVA, using the untransformed percentage premiums, showed a statistically significant magnitude effect that was attenuated by the percent frame. The medians and interquartile ranges are presented in Table S1. The main effect of principal amount was statistically significant, reflecting a magnitude effect, such that percentage premiums were greater for the small principal than the large principal (*F*(1, 204) = 103.67, *p* < .001). The main effect was qualified by a statistically significant interaction between principal and frame (*F*(1, 204) = 42.20, *p* < .001). Using the Wilcoxon Signed-Rank Test with continuity correction, we see that the magnitude effect—that is, the difference between the percentage premiums for the small and large principal amounts—was greater for participants in the currency frame (*Z* = -5.84, *p* < .001, *r* = 0.41) than for participants in the percent frame (*Z* = -2.66, *p* = .008, *r* = 0.19). Figure S1 visually represents the results for the percentage premiums in the loss domain.

# Study 2

We conducted a second study to address the inconclusive results of Study 1 in the gain domain. Given that a small number of participants in Study 1 used the text box at the end of the survey to comment that they found the questions difficult to understand, indicating some confusion, we reconsidered the wording of the questions. Improved clarity would reduce confusion and thus noise in the data, thereby increasing statistical power. Moreover, we increased the sample size by about 30%. In Study 2, we once again tested the attenuation hypothesis in the gain domain, predicting that the percent frame would attenuate the magnitude effect.

## Method

All methods and procedural details were very similar to Study 1, except that we focused only on the gain domain and collected no data for the loss domain.

### Participants

In addition to improving the clarity of our questions, we aimed for a larger sample size to increase statistical power for the same effect sizes, though we did not conduct power analyses for this study. We aimed for a total of 260 participants (130 per between-subjects condition). After pre-registered exclusions, we ended with a sample size of 261 participants recruited using the Prolific platform and the same prescreening criteria as in Study 1 (to include only participants with U.K. residency and who were fluent in English), excluding those who participated in Study 1; 162 female, 97 male, and 2 other, mean age 36.7 years (*SD* = 10.7).

### Design

In Study 2, we conducted an experiment with 4 conditions: We used a 2 (principal amount: small vs large) by 2 (frame: currency vs percent) mixed design. Principal amount was a within-subjects factor and frame was a between-subjects factor.

### Procedure

The procedure was exactly the same as for Study 1 except that the wording of the instructions and questions were slightly changed. For the precise details of the wording of the instructions and questions, we refer readers to the Supplemental materials (see under heading, “Study 2 – Procedure & Pre-Registered Dependent Variables”). After completing the intertemporal choice task, we asked participants their sex, year of birth, education level, to “Please rate the clarity of the questions of this survey” (from 1 = *not clear at all*, to 7 = *very clear*, without labelling the points in between; *M* = 5.51, *SD* = 1.70), and an open text box to provide comments on the clarity of the questions.

### Measures

The outcome variable for Study 2 was the same as for Study 1, the percentage premium.

## Results

To test the hypothesis that the magnitude effect would be attenuated by the percent frame, we used a nonparametric ANOVA, with principal amount as the within-subjects factor and framing as the between-subjects factor, and percentage premiums as the dependent variable. Table S1 presents the descriptive statistics. We observed an overall magnitude effect. That is, the percentage premium was greater for the small principal than for the large principal (see Table S1), as indicated by the statistically significant main effect of principal amount (*F*(1, 259) = 131.66, *p* < .0001). Critically, the main effect of principal amount was qualified by a statistically significant interaction with the framing factor (*F*(1, 259) = 11.46, *p* = .0008). Using the Wilcoxon Signed-Rank Test with continuity correction to probe into the interaction further, we found that the magnitude effect was greater for participants in the currency frame (*Z* = -6.97, *p* < .0001, *r* =.43) than for participants in the percent frame (*Z* = -5.73, *p* < .0001, *r* = .35). Figure S2 visually represents the results. The results showed support for the attenuation hypothesis: The magnitude effect was attenuated by the percent frame.

*
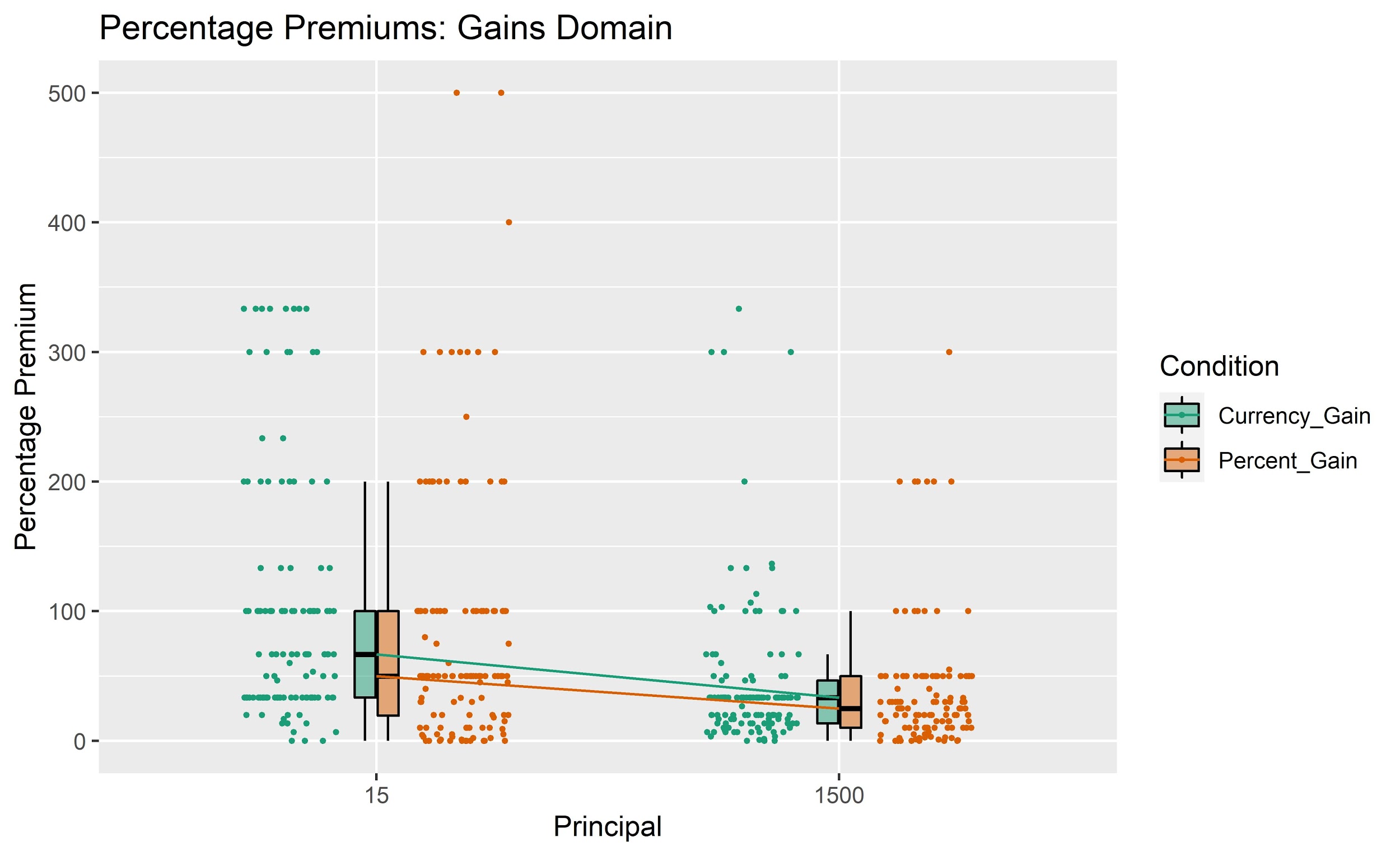
*

*Figure S2*. Study 2, Gain Domain. Density plot and median boxplot. The lower and upper hinges of the box represent the 25th and 75th percentiles, respectively, the distance between them being the interquartile range (IQR). The upper whisker extends to the highest percentage premium that is no further from the upper hinge than 1.5*IQR, and the lower whisker extends to the lowest percentage premium that is no further than 1.5*IQR from the lower hinge. The line inside each box represents the median of the percentage premium. The coloured lines connect the medians of the percentage premium from the small to large principal amounts. A downward slope suggests a magnitude effect; the steeper the slope the larger the magnitude effect. Note that 4 observations fall above the y-axis upper limit: 1 from the large principal percent frame, and 3 from the small principal currency frame.

# Study 3

Although the results of Studies 1 and 2 largely supported the attenuation hypothesis, both studies used the same small and large principal amounts. We thus conducted a third study with different small and large principal amounts. In addition, we aimed to replicate the results for the loss domain. In Study 3, we tested the same attenuation hypothesis in the gain and loss domains with new principal amounts. We used the same analyses as in the previous studies.

## Method

All methods and procedural details were very similar to Studies 1 and 2, with the only differences being shorter and more precise instructions (full details provided in the Supplemental Materials) and different principals: the small principal amount for this study was £45 and the large principal was £1,300.

### Participants

We aimed for a total of 600 participants. After pre-registered exclusions (i.e., those who failed to complete the survey and the second response of those who were recorded twice), we ended with a sample size of 593 participants. We used the same prescreening criteria as Studies 1 and 2, excluding those who participated in the earlier studies. Participants were 376 females, 215 males, and 2 other, mean age 36.9 years (*SD* = 13).

### Design, Procedure, and Measures

For Study 3 we conducted an experiment with 8 conditions using an identical design to Study 1. The procedure was the same as for Study 1 except that the two principal amounts were presented on separate pages. The outcome variable was, as in the previous studies, the percentage premium.

## Results

Table S1 presents the descriptive statistics. We conducted an exploratory 3-way nonparametric ANOVA with domain, principal amount, and framing as the factors. Consistent with Study 1, the magnitude effect was smaller in the domain of losses than in the domain of gains, as indicated by the 2-way interaction between domain and principal amount (*F*(1, 589) = 30.98, *p* < .0001). As can be seen in Table S1 (the “Total” rows), the percentage premium for the small principal was larger than the percentage premium for the large principal (i.e., magnitude effect), and this magnitude effect was statistically significantly smaller in the loss domain than in the gain domain.

### Gain Domain

We observed an overall magnitude effect in the gain domain. The percentage premium was greater for the small principal than for the large principal (see Table S1), as indicated by the statistically significant main effect of principal amount (*F*(1, 300) = 94.44, *p* < .0001). Critically, the main effect of principal amount was qualified by a statistically significant interaction with the framing factor (*F*(1, 300) = 18.54, *p* < .0001). The magnitude effect was greater for participants in the currency frame (*Z* = -7.91, *p* < .0001, *r* =.45) than for those in the percent frame (*Z* = -4.43, *p* < .0001, *r* = .26). The attenuation hypothesis was thus supported. Figure S3 visually represents the results.

### Loss Domain

There was a magnitude effect in the loss domain, such that the percentage premium was larger for the small principal than for the large principal (see Table S1), as indicated by a statistically significant main effect of principal amount (*F*(1, 289) = 4.38, *p* = .0372). Although the magnitude effect was greater for participants in the currency frame (*Z* = -5.11, *p* < .0001, *r* =.29) than for those in the percent frame (*Z* = -1.24, *p* = .2168, *r* = .08), this difference was not statistically significant. The interaction between the principal amount and framing was not statistically significant (*F*(1, 289) = 0.31, *p* = .5807). Figure S3 visually represents the results. The attenuation hypothesis was in this case not supported.


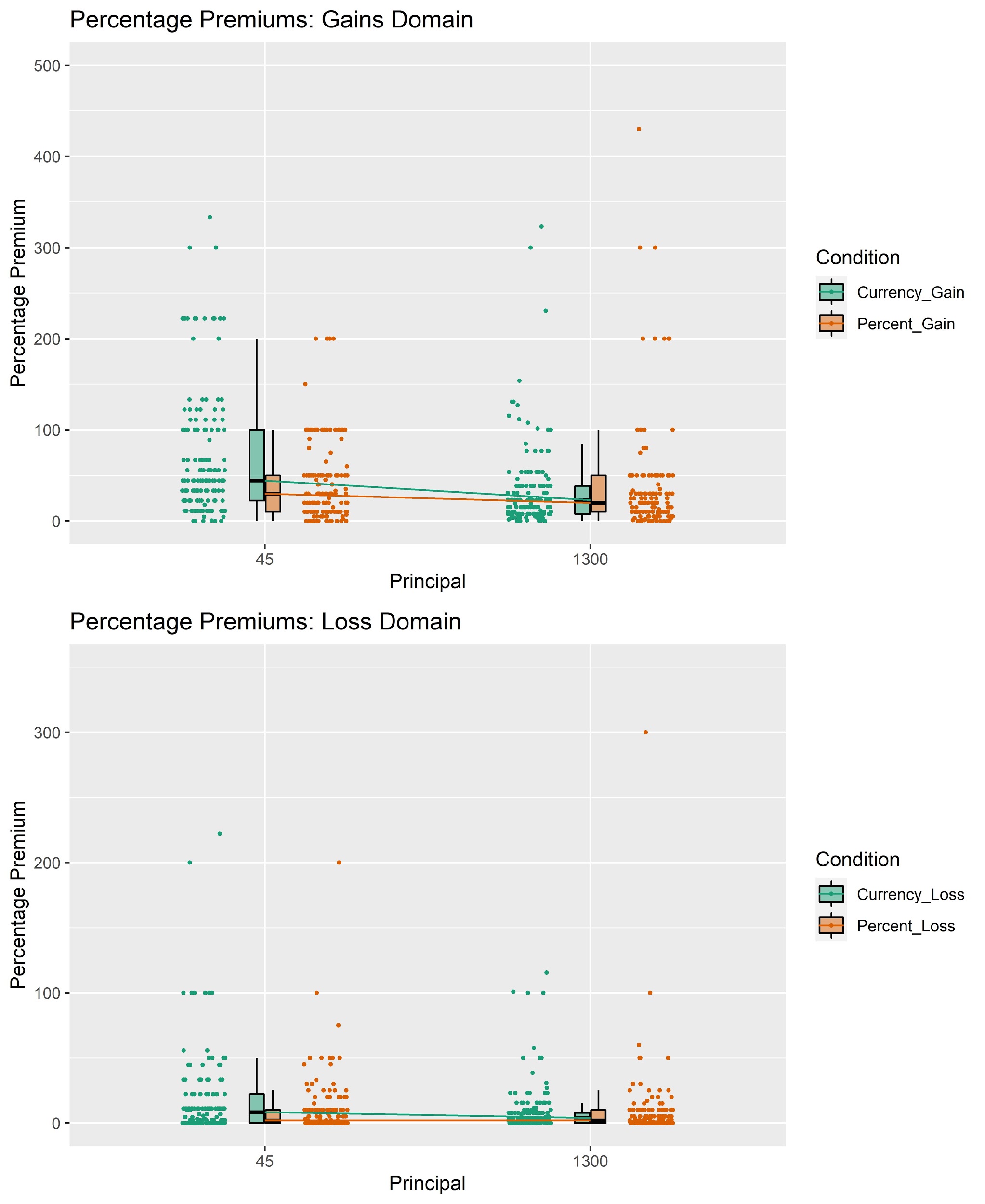


*Figure S3*. Study 3. Upper panel displays results for gains and the lower panel for losses. Density plot and median boxplot. The lower and upper hinges of the box represent the 25th and 75th percentiles, respectively, the distance between them being the interquartile range (IQR). The upper whisker extends to the highest percentage premium that is no further from the upper hinge than 1.5*IQR, and the lower whisker extends to the lowest percentage premium that is no further than 1.5*IQR from the lower hinge. The line inside each box represents the median of the percentage premium. The coloured lines connect the medians of the percentage premium from the small to large principal amounts. A downward slope suggests a magnitude effect; the steeper the slope the larger the magnitude effect. Note that 1 observation from the small principal currency frame falls above the y-axis upper limit of the gain domain; 2 observations from the large principal percent frame fall above the y-axis upper limit of the loss domain.

# Study 4

The results of Study 3 were inconclusive for the loss domain. Although the magnitude effect was descriptively larger in the currency frame than in the percent frame, this difference was not statistically significant (i.e., nonsignificant interaction). This may be due to a Type 2 error, where a true effect has not been detected. In addition, it could be that the difference in the size of the magnitude effect between the currency and percent frames was smaller than in Study 1, making it more difficult to statistically be detected. The cause of this may be varied. We speculated that one reason could be because the small principal amount in Study 3 was considerably (i.e., 3 times) larger than the small principal in Study 1, where we did find a statistically significant attenuation. We therefore conducted another study for the loss domain using a small principal amount that was nearer to the small principal in Study 1, while changing the large principal amount considerably.

## Method

All methods and procedural details were identical to Study 3 except we only collected data for the loss domain and the principal amounts were changed. The small principal amount was £12 and the large principal was £750. Full details are provided in the Supplemental Materials.

### Participants

We aimed for a total of 300 participants. After excluding participants who failed to complete the survey (whose responses were not recorded) and the second response of those who appeared twice in the dataset, we ended with a sample size of 298 participants. We used the same prescreening criteria as Studies 1, 2, and 3, excluding those who participated in the earlier studies. Participants were 199 females, 98 males, and 1 other, mean age 37.2 years (*SD* = 13.2).

### Design, Procedure, and Measures

For Study 4 we conducted an experiment with four conditions. We used a 2 (principal amount: small vs large) by 2 (frame: currency vs percent) mixed design. Principal amount was a within-subjects factor and frame was a between-subjects factor. The procedure was the same as for Study 3. The outcome variable was, as in the previous studies, the percentage premium.

## Results

Table S1 presents the descriptive statistics. We observed an overall magnitude effect such that the percentage premium was greater for the small principal than for the large principal (see Table S1), as indicated by the statistically significant main effect of principal amount (*F*(1, 296) = 41.14, *p* < .0001). Critically, this was qualified by a statistically significant interaction with the framing factor (*F*(1, 296) = 29.84, *p* < .0001). The magnitude effect was greater for participants in the currency frame (*Z* = -6.82, *p* < .0001, *r* =.39) than for those in the percent frame (*Z* = -1.05, *p* = .2954, *r* = .06). Figure S4 visually represents the results. Therefore, the attenuation hypothesis was supported in the loss domain.


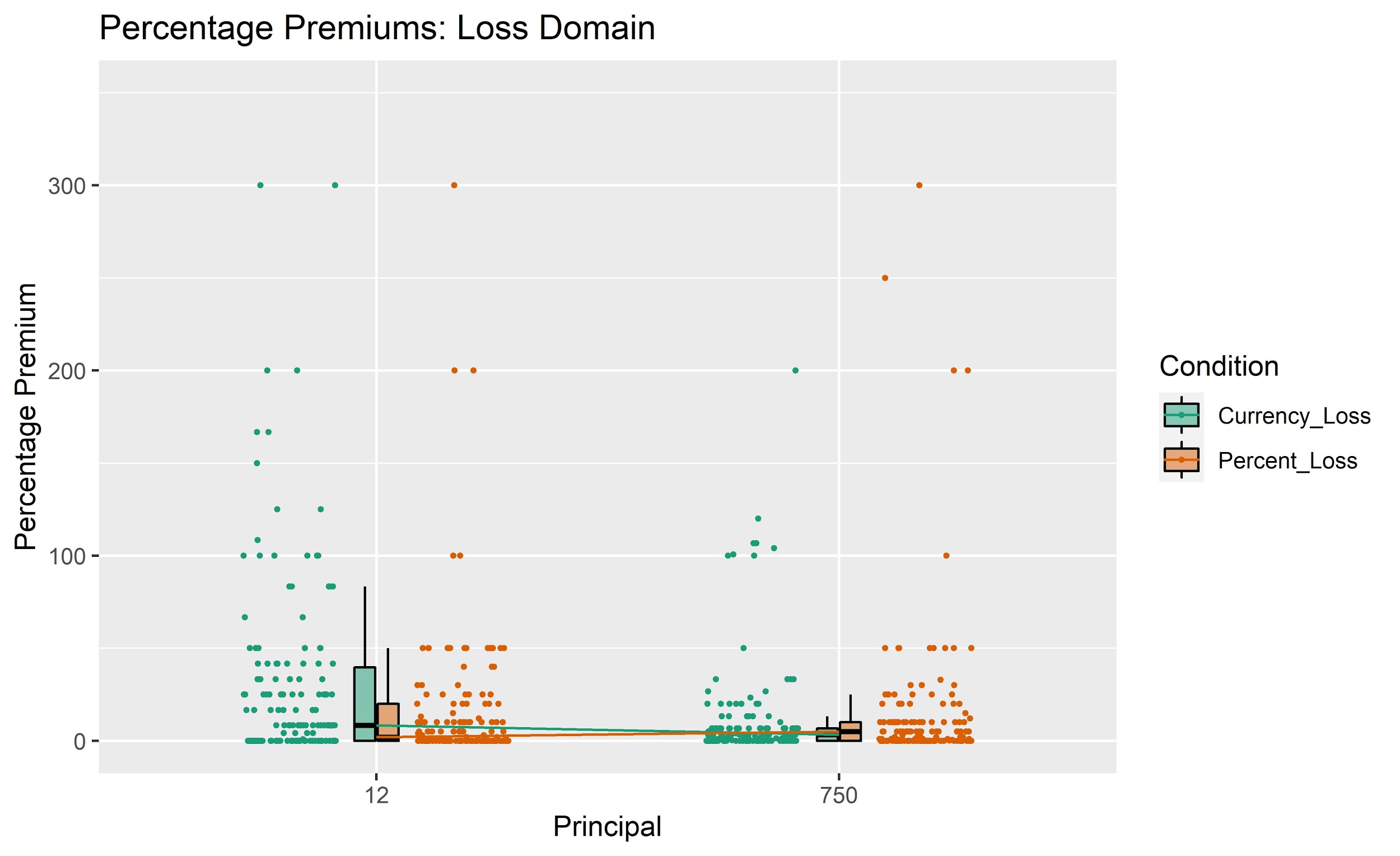


*Figure S4*. Study 4, loss domain. Density plot and median boxplot. The lower and upper hinges of the box represent the 25th and 75th percentiles, respectively, the distance between them being the interquartile range (IQR). The upper whisker extends to the highest percentage premium that is no further from the upper hinge than 1.5*IQR, and the lower whisker extends to the lowest percentage premium that is no further than 1.5*IQR from the lower hinge. The line inside each box represents the median of the percentage premium. The coloured lines connect the medians of the percentage premium from the small to large principal amounts. A downward slope suggests a magnitude effect; the steeper the slope the larger the magnitude effect. Note that 1 observation from the small principal currency frame falls above the y-axis upper limit and 1 observation from the large principal percent frame falls above the y-axis upper limit.

# References

See main manuscript for full reference list

1. We first pre-registered the details for Study 1 here <https://osf.io/aphyq>, but decided to add an outlier exclusion rule prior to having collected data. Thus, we ended up with two almost identical pre-registrations for Study 1. The data and analysis code for both studies can be found on the Open Science Framework (<https://osf.io/qgxpf/>). We report how we determined our sample size, all data exclusions, all manipulations, and all measures in both studies. Notably, although we pre-registered our intention to use exponential and hyperbolic discount rates as the dependent variables for both studies, the results of which are both fully reported in the Supplemental materials (<https://osf.io/myw37/>), in the main text we report the results for analyses using a calculation that is more intuitively understandable—the percentage premium. The conclusions and inferences are unchanged. For Study 1 and 2, we pre-registered our intention to exclude participants who completed the task in under 60 and 40 seconds, respectively, but no participants did so. Furthermore, for Study 1, we intended to log-transform the outcome variables, if they were highly skewed (indeed they were), before conducting the parametric ANOVAs (in addition to conducting nonparametric ANOVAs). However, the log-transformations resulted in non-random data loss, bringing the validity of these analyses into question. We report all of these analyses, including a log-transformation approach that produces no data loss, in the Supplemental Materials. In the main text, however, for the purposes of brevity, we report the results of the nonparametric analyses on the untransformed outcome variable. Inferences remain largely unchanged regardless of the analytic approach. [↑](#footnote-ref-2)
