# Supplementary material for: Percent framing attenuates the magnitude effect in a preference-matching task of intertemporal choice
Source: PLoS One. 2022 Jan 24;17(1):e0262620. doi: 10.1371/journal.pone.0262620 (PMC8786190; doi:10.1371/journal.pone.0262620)
Supplement: Peer review history

# Peer review: Decision Letter 0

**DOI:** 10.1371/journal.pone.0262620.r001

**Authors:** David V. Smith

1 Dec 2021

PONE-D-21-31599Percent framing attenuates the magnitude effect in a preference-matching task of intertemporal choicePLOS ONE

Dear Dr. Anvari,

Thank you for submitting your manuscript to PLOS ONE. After careful consideration, we feel that it has merit but does not fully meet PLOS ONE’s publication criteria as it currently stands. Therefore, we invite you to submit a revised version of the manuscript that addresses the points raised during the review process.

Two expert reviewers have evaluated your manuscript. Although both reviewers highlight strengths of your submission, there are also some weaknesses that should be addressed in a revision. For example, Reviewer 2 raises issues regarding the statistical analyses. In addition, Reviewer 1 raises two important issues about the contextualization and interpretation of the results. The full reviewer reports are appended below. 

Please submit your revised manuscript by Jan 15 2022 11:59PM. If you will need more time than this to complete your revisions, please reply to this message or contact the journal office at plosone@plos.org. When you're ready to submit your revision, log on to https://www.editorialmanager.com/pone/ and select the 'Submissions Needing Revision' folder to locate your manuscript file.

Please include the following items when submitting your revised manuscript:

- A rebuttal letter that responds to each point raised by the academic editor and reviewer(s). You should upload this letter as a separate file labeled 'Response to Reviewers'.
- A marked-up copy of your manuscript that highlights changes made to the original version. You should upload this as a separate file labeled 'Revised Manuscript with Track Changes'.
- An unmarked version of your revised paper without tracked changes. You should upload this as a separate file labeled 'Manuscript'.

If you would like to make changes to your financial disclosure, please include your updated statement in your cover letter. Guidelines for resubmitting your figure files are available below the reviewer comments at the end of this letter.

If applicable, we recommend that you deposit your laboratory protocols in protocols.io to enhance the reproducibility of your results. Protocols.io assigns your protocol its own identifier (DOI) so that it can be cited independently in the future. For instructions see: https://journals.plos.org/plosone/s/submission-guidelines#loc-laboratory-protocols. Additionally, PLOS ONE offers an option for publishing peer-reviewed Lab Protocol articles, which describe protocols hosted on protocols.io. Read more information on sharing protocols at https://plos.org/protocols?utm_medium=editorial-email&utm_source=authorletters&utm_campaign=protocols.

We look forward to receiving your revised manuscript.

Kind regards,

David V. Smith, Ph.D.

Academic Editor

PLOS ONE

Journal Requirements:

When submitting your revision, we need you to address these additional requirements.

1. Please ensure that your manuscript meets PLOS ONE's style requirements, including those for file naming. The PLOS ONE style templates can be found at 

https://journals.plos.org/plosone/s/file?id=wjVg/PLOSOne_formatting_sample_main_body.pdf and 

https://journals.plos.org/plosone/s/file?id=ba62/PLOSOne_formatting_sample_title_authors_affiliations.pdf

2. Thank you for stating the following financial disclosure: 

"D.M. received funding from Danish Council for Independent Research DFF – 7015 – 00050, which was used for the studies.

F.A. was supported by funding from the European Union’s Horizon 2020 research and innovation programme under the Marie Sklodowska-Curie grant agreement No 883785."

Please state what role the funders took in the study.  If the funders had no role, please state: "The funders had no role in study design, data collection and analysis, decision to publish, or preparation of the manuscript." 

If this statement is not correct you must amend it as needed. 

Please include this amended Role of Funder statement in your cover letter; we will change the online submission form on your behalf.

3. Your ethics statement should only appear in the Methods section of your manuscript. If your ethics statement is written in any section besides the Methods, please move it to the Methods section and delete it from any other section. Please ensure that your ethics statement is included in your manuscript, as the ethics statement entered into the online submission form will not be published alongside your manuscript. 

[Note: HTML markup is below. Please do not edit.]

Reviewers' comments:

Reviewer's Responses to Questions

**Comments to the Author**

1. Is the manuscript technically sound, and do the data support the conclusions?

The manuscript must describe a technically sound piece of scientific research with data that supports the conclusions. Experiments must have been conducted rigorously, with appropriate controls, replication, and sample sizes. The conclusions must be drawn appropriately based on the data presented. 

Reviewer #1: Yes

Reviewer #2: Partly

**********

2. Has the statistical analysis been performed appropriately and rigorously? 

Reviewer #1: Yes

Reviewer #2: Yes

**********

3. Have the authors made all data underlying the findings in their manuscript fully available?

The PLOS Data policy requires authors to make all data underlying the findings described in their manuscript fully available without restriction, with rare exception (please refer to the Data Availability Statement in the manuscript PDF file). The data should be provided as part of the manuscript or its supporting information, or deposited to a public repository. For example, in addition to summary statistics, the data points behind means, medians and variance measures should be available. If there are restrictions on publicly sharing data—e.g. participant privacy or use of data from a third party—those must be specified.

Reviewer #1: Yes

Reviewer #2: Yes

**********

4. Is the manuscript presented in an intelligible fashion and written in standard English?

PLOS ONE does not copyedit accepted manuscripts, so the language in submitted articles must be clear, correct, and unambiguous. Any typographical or grammatical errors should be corrected at revision, so please note any specific errors here.

Reviewer #1: Yes

Reviewer #2: Yes

**********

5. Review Comments to the Author

Please use the space provided to explain your answers to the questions above. You may also include additional comments for the author, including concerns about dual publication, research ethics, or publication ethics. (Please upload your review as an attachment if it exceeds 20,000 characters)

Reviewer #1: This article reports the results of four studies examining whether presenting options in percentages as opposed to amounts attenuates the magnitude effect in intertemporal choice. In four studies, the authors use the preference matching approach to assess the magnitude effect in intertemporal choice. They use large and small amounts in the domains of both gains and losses, soliciting preferences either in amounts or in percentages. They demonstrate that the size of the magnitude effect is smaller when the solicitation occurs in percentages as opposed to amounts. These findings are discussed in the context of existing theories of intertemporal choice, most prominently DRIFT.

The paper is clearly written and the results are consistent across all four experiments. The authors also do a good job clarifying the contribution of their work. They rightly acknowledge that the attenuation of the magnitude effect by percentage elicitations has already been demonstrated using the binary choice task in intertemporal choice. They seek therefore to extend this finding to the preference matching approach and to the loss domain.

Because of non-normal characteristics of the data distributions, the authors report their results using effect sizes rather than traditional inferential statistics. This approach is unusual, but valid in the current circumstances given the non-normal data distributions. Indeed, looking across the studies in Table 1, the findings are remarkably consistent and the 95% CIs support the authors’ conclusions. While it is unusual to read a paper with no p-values for comparisons between conditions, the approach is warranted in the present case.

I would like to see the discussion of the results updated in two important ways. First, the decisions presented to participants are all hypothetical, and participants do not experience the outcomes of their decisions. It would have been preferable to have the decisions be incentive-compatible, with participants experiencing the outcomes they select. However, I appreciate the fact that this is difficult to do in practice (particularly for large magnitudes and losses). I think it would still be appropriate for the authors to acknowledge as a limitation that the decisions are all hypothetical in nature. Second, the authors consistently discuss their findings as supporting DRIFT. By the same token, however, they support other time-as-attribute models of intertemporal choice that have been proposed, including ITCH (Ericson et al., 2015). While the ITCH model is cited, it is not acknowledged directly in the text to the same degree that DRIFT is. I was encourage the authors to be more even-handed in their discussion, mentioning that both DRIFT and ITCH are supported by the current findings.

Overall, this was an interesting paper and the results are clear.

Reviewer #2: Anvari and coauthors present a study testing whether the magnitude effect in intertemporal choice is attenuated when attention is drawn to the relative difference, rather than the absolute difference, in values between the options. Evidence in support of this theory suggests that the magnitude effect arises due to psychological factors, as postulated by the DRIFT model, rather than by some idiosyncratic economic factor. While this effect has been shown before, as the authors point out, the authors tested for this effect using a very different procedure and tested, for the first time, for this effect in the domain of losses.

Overall, I find the paper well-written and the analyses are clear. They find consistent results across four studies that appear large, and I was impressed by the control analyses, including one ruling out a preference for choice consistency as a driver of the effect. However, I have major concerns about the lack of statistical analyses and the lack of depictions of the data.

1) The authors argue that the unequal variances and skew between their conditions precludes statistical testing, and instead adopt a “meta-analytical perspective” of qualitatively comparing effect size metrics. It is clear that there is a large skew in the data given the large differences between the means and medians, so I am sympathetic to the authors’ desire to avoid inappropriate statistical tests. However, why does unequal variance and skew preclude nonparamatretic testing? If the authors are convinced that these tests are not appropriate, could they at least do a formal meta-analytical statistical test on the effect size across their studies? Without any statistical tests at all, then I think a caveat that the results are descriptive would be needed in the abstract.

2) The data are only depicted as differences of means, and in tables. I would like to see plots of the subjects’ raw preferences by condition (pounds or percentages, for large and small magnitudes), at least in the Appendix. That could help an interested reader what is going on to drive the large skew and variance effects.

3) It seems possible that many people do not understand the concept of percent difference well. Is it possible that the reduction of the magnitude effect in the percent difference conditions comes from participant confusion about what that quantity means? Subjects may not be aware of what dollar amounts are at stake for a chosen percentage.

4) I am confused about the negative magnitude effect in some loss conditions. While technically this means percent framing reduces the magnitude effect, in one case (study 3) there is a much larger effect of magnitude in the percent framing condition, but in the opposite direction. I would like to see these results unpacked and explained in more detail, given that the loss domain is a principle novel advance of the study.

Minor:

1) The first sentence of the abstract defines the magnitude effect, but the definition that follows describes temporal discounting generally, not the magnitude effect.

2) The table legends in the main text report there being medians and nonparametric standardized effect size metrics, but these refer to tables in the Appendix.

**********

6. PLOS authors have the option to publish the peer review history of their article (what does this mean?). If published, this will include your full peer review and any attached files.

If you choose “no”, your identity will remain anonymous but your review may still be made public.

**Do you want your identity to be public for this peer review?** For information about this choice, including consent withdrawal, please see our Privacy Policy.

Reviewer #1: No

Reviewer #2: No

[NOTE: If reviewer comments were submitted as an attachment file, they will be attached to this email and accessible via the submission site. Please log into your account, locate the manuscript record, and check for the action link "View Attachments". If this link does not appear, there are no attachment files.]

While revising your submission, please upload your figure files to the Preflight Analysis and Conversion Engine (PACE) digital diagnostic tool, https://pacev2.apexcovantage.com/. PACE helps ensure that figures meet PLOS requirements. To use PACE, you must first register as a user. Registration is free. Then, login and navigate to the UPLOAD tab, where you will find detailed instructions on how to use the tool. If you encounter any issues or have any questions when using PACE, please email PLOS at figures@plos.org. Please note that Supporting Information files do not need this step.

# Author comment: Author response to Decision Letter 0

**DOI:** 10.1371/journal.pone.0262620.r002

9 Dec 2021

Dear Dr. Smith,

Thank you for your efforts in editing our manuscript and thanks also to the reviewers. As suggested by the decision letter, below we present our responses to each point raised by the reviewers. First, we present the reviewer’s point, then our response, and so on. In our responses, we note the page numbers in the manuscript where we’ve made changes or additions. These page numbers are in reference to the “Revised Manuscript with Track Changes”. 

Furthermore, we’ve updated the funding disclosure to include the statement, “The funders had no role in study design, data collection and analysis, decision to publish, or preparation of the manuscript.”.

If there is anything else you would like from us, please do not hesitate to contact us.

We hope that you agree that the reviewers’ points have been thoroughly addressed, and well.

We look forward to hearing further from you.

Kind regards,

Farid Anvari and the authorship team.

Reviewer 1 

General point

This article reports the results of four studies examining whether presenting options in percentages as opposed to amounts attenuates the magnitude effect in intertemporal choice. In four studies, the authors use the preference matching approach to assess the magnitude effect in intertemporal choice. They use large and small amounts in the domains of both gains and losses, soliciting preferences either in amounts or in percentages. They demonstrate that the size of the magnitude effect is smaller when the solicitation occurs in percentages as opposed to amounts. These findings are discussed in the context of existing theories of intertemporal choice, most prominently DRIFT.

The paper is clearly written and the results are consistent across all four experiments. The authors also do a good job clarifying the contribution of their work. They rightly acknowledge that the attenuation of the magnitude effect by percentage elicitations has already been demonstrated using the binary choice task in intertemporal choice. They seek therefore to extend this finding to the preference matching approach and to the loss domain.

*** Response. We thank the reviewer for the kind words. 

Point 1

Because of non-normal characteristics of the data distributions, the authors report their results using effect sizes rather than traditional inferential statistics. This approach is unusual, but valid in the current circumstances given the non-normal data distributions. Indeed, looking across the studies in Table 1, the findings are remarkably consistent and the 95% CIs support the authors’ conclusions. While it is unusual to read a paper with no p-values for comparisons between conditions, the approach is warranted in the present case.

*** Response. We agree that not doing formal significance tests with p-values was unconventional. We weren’t sure about doing this ourselves. But we also agree that the data were such that this was the best solution, given the distributions of the data.

Point 2

I would like to see the discussion of the results updated in two important ways. First, the decisions presented to participants are all hypothetical, and participants do not experience the outcomes of their decisions. It would have been preferable to have the decisions be incentive-compatible, with participants experiencing the outcomes they select. However, I appreciate the fact that this is difficult to do in practice (particularly for large magnitudes and losses). I think it would still be appropriate for the authors to acknowledge as a limitation that the decisions are all hypothetical in nature. 

*** Response. We certainly agree with the reviewer that this is a limitation of the work we present in this paper. We had mentioned this limitation, albeit by extremely briefly mentioning that future work can examine the generalizability of the results in the presence of real stakes. To make this limitation clearer, we’ve added the following passage (see p. 29):

“In particular, the decisions presented to participants in the studies were always hypothetical, without participants experiencing the outcomes of their decisions. Future research may examine the hypotheses using real financial incentives, though this may not be practical with gains of large magnitude and for losses. “ 

Point 3

Second, the authors consistently discuss their findings as supporting DRIFT. By the same token, however, they support other time-as-attribute models of intertemporal choice that have been proposed, including ITCH (Ericson et al., 2015). While the ITCH model is cited, it is not acknowledged directly in the text to the same degree that DRIFT is. I was encourage the authors to be more even-handed in their discussion, mentioning that both DRIFT and ITCH are supported by the current findings.

*** Response. The reviewer is of course correct. We’ve now added to the relevant sections throughout the manuscript to indicate that not only is the DRIFT model supported, but also the ITCH model. See pp. 5, 6, 8-10, 27, and 29.

Overall, this was an interesting paper and the results are clear.

*** Response. We thank the reviewer for the kind words.

Reviewer 2

General Point

Anvari and coauthors present a study testing whether the magnitude effect in intertemporal choice is attenuated when attention is drawn to the relative difference, rather than the absolute difference, in values between the options. Evidence in support of this theory suggests that the magnitude effect arises due to psychological factors, as postulated by the DRIFT model, rather than by some idiosyncratic economic factor. While this effect has been shown before, as the authors point out, the authors tested for this effect using a very different procedure and tested, for the first time, for this effect in the domain of losses.

Overall, I find the paper well-written and the analyses are clear. They find consistent results across four studies that appear large, and I was impressed by the control analyses, including one ruling out a preference for choice consistency as a driver of the effect. 

*** Response. We thank the reviewer for the kind words.

Point 1

However, I have major concerns about the lack of statistical analyses and the lack of depictions of the data.

1) The authors argue that the unequal variances and skew between their conditions precludes statistical testing, and instead adopt a “meta-analytical perspective” of qualitatively comparing effect size metrics. It is clear that there is a large skew in the data given the large differences between the means and medians, so I am sympathetic to the authors’ desire to avoid inappropriate statistical tests. However, why does unequal variance and skew preclude nonparamatretic testing? If the authors are convinced that these tests are not appropriate, could they at least do a formal meta-analytical statistical test on the effect size across their studies? Without any statistical tests at all, then I think a caveat that the results are descriptive would be needed in the abstract.

*** Response. We refer the reviewer to Reviewer 1’s point 1. We think that the parametric tests’ assumptions have been violated too strongly for them to be unbiased. That is, the skew combined with unequal variances creates obvious problems for parametric statistical tests. 

In addition, the non-parametric alternative is also problematic here because of heteroscedasticity. In relation to this, the possibility of using a Mann-Whitney-Wilcoxon (MWW) test is not recommended because of the following two reasons. First, the MWW is truly a test of medians, but only if it is assumed that the two populations being compared have identical shapes (and thus variances) and that they differ only by a shift alternative. This is not the case for our data, so that the measure actually tested by the MWW is Pr(X1 < X2) + Pr(X1 = X2)/2, where X1 and X2 are random observations from the two groups being compared. Differently than the medians, such measure is not straightforward to present and can create confusion to the reader (on this, see the nice paper: George W. Divine, H. James Norton, Anna E. Barón & Elizabeth Juarez-Colunga (2017): The Wilcoxon–Mann–Whitney Procedure Fails as a Test of Medians, The American Statistician, DOI: 10.1080/00031305.2017.1305291). The second reason is that one major limitation of the MWW test is that the type I error is inflated in a situation of inequality of variances (even though the sample size is equal; see for example D. W. Zimmerman (2003) A Warning About the Large-Sample Wilcoxon-Mann-Whitney Test, Understanding Statistics, 2:4, 267-280, DOI: 10.1207/S15328031US0204_03).

Nonetheless, we agree with the reviewer that a caveat is necessary. In the main text we have already included just such a caveat by thoroughly describing why we didn’t use the parametric tests and for how we instead use the descriptive statistics to examine whether the results support the hypotheses (see section under “Results”, pp. 18-19). We’ve now included a similar caveat in the abstract as well:

“The data were heavily skewed and the assumption of equal variances were violated. Therefore, in place of parametric statistical tests, we calculated and interpreted parametric and nonparametric standardized and unstandardized effect size estimates and their confidence intervals.”

Point 2

2) The data are only depicted as differences of means, and in tables. I would like to see plots of the subjects’ raw preferences by condition (pounds or percentages, for large and small magnitudes), at least in the Appendix. That could help an interested reader what is going on to drive the large skew and variance effects.

*** Response. The data are also depicted as differences of medians (see Tables A1 and A2, in Appendix). We agree with the reviewer that figures would be useful for illustrative purposes. We’ve now therefore included a figure showing the distribution of responses across all 4 studies. These are presented in the Appendix. In the main text, we introduce these figures (see p. 20), “Figs A1-A4 in the Appendix show the distribution of responses of the percentage premium, a, for the small and large principal amounts across the currency and percent conditions in Studies 1-4, respectively.”.

Point 3

3) It seems possible that many people do not understand the concept of percent difference well. Is it possible that the reduction of the magnitude effect in the percent difference conditions comes from participant confusion about what that quantity means? Subjects may not be aware of what dollar amounts are at stake for a chosen percentage.

*** Response. It may be the case that participants aren’t aware, in the moment, of the dollar amount at stake for the chosen percentage. However, this is a feature of the design. Indeed, the DRIFT model (and ITCH) propose that the percent frame reduces the magnitude effect precisely because it diverts people’s attention away from the absolute difference (i.e., the dollar amounts) and focuses people more towards the relative difference (i.e., the percentage difference; see p. 5). 

With regards to whether participants understood the task in general, we had every participant respond to two practice preliminary questions before the main task (see pp. 12-14 for description of the practice questions). Participants had to answer these correctly before they could continue to the main task. This was designed so that people would participate only after having indicated that they understood the task.

Point 4

4) I am confused about the negative magnitude effect in some loss conditions. While technically this means percent framing reduces the magnitude effect, in one case (study 3) there is a much larger effect of magnitude in the percent framing condition, but in the opposite direction. I would like to see these results unpacked and explained in more detail, given that the loss domain is a principle novel advance of the study.

*** Response. We acknowledge the reviewer’s point. Namely, Studies 3 and 4, but not Study 1, show that in the losses domain, the percent frame may have created a reverse magnitude effect. Although we hesitate to interpret these findings too strongly, we now discussed them in more depth (see p. 28):

“Nonetheless, in Studies 3 and 4, but not Study 1, there were potential reverse magnitude effects in the percent frame of the losses domain as indicated by the negative sign of the effect sizes in Tables 1 and 2. Thus, it may be the case that people prefer to pay larger debts sooner when the choice is framed such that attention is directed to the relative difference between paying now and paying later. However, we hesitate to draw strong conclusions or interpretations about this finding because the effect sizes for a possible reverse magnitude effect had wide confidence intervals that included positive effect sizes. Moreover, the estimate for the effect in Study 1 was positive, though the confidence intervals included negative effect sizes. Hence, it’s unclear whether the reversal in Studies 3 and 4 are spurious. Future research may examine this potential reversal, perhaps with larger sample sizes to obtain more precise estimates.”

Minor points

Minor:

1) The first sentence of the abstract defines the magnitude effect, but the definition that follows describes temporal discounting generally, not the magnitude effect.

*** Response. We thank the reviewer for pointing this out. We’ve corrected the beginning of the abstract, defining temporal discounting first, and then the magnitude effect in the next sentence.

2) The table legends in the main text report there being medians and nonparametric standardized effect size metrics, but these refer to tables in the Appendix.

*** Response. We thank the reviewer for noticing this. We’ve corrected Table 2 now.

Attachment Submitted filename: Response to Reviewers.docx Click here for additional data file.

# Peer review: Decision Letter 1

**DOI:** 10.1371/journal.pone.0262620.r003

**Authors:** David V. Smith

31 Dec 2021

Percent framing attenuates the magnitude effect in a preference-matching task of intertemporal choice

PONE-D-21-31599R1

Dear Dr. Anvari,

We’re pleased to inform you that your manuscript has been judged scientifically suitable for publication and will be formally accepted for publication once it meets all outstanding technical requirements.

Within one week, you’ll receive an e-mail detailing the required amendments. When these have been addressed, you’ll receive a formal acceptance letter and your manuscript will be scheduled for publication.

An invoice for payment will follow shortly after the formal acceptance. To ensure an efficient process, please log into Editorial Manager at http://www.editorialmanager.com/pone/, click the 'Update My Information' link at the top of the page, and double check that your user information is up-to-date. If you have any billing related questions, please contact our Author Billing department directly at authorbilling@plos.org.

If your institution or institutions have a press office, please notify them about your upcoming paper to help maximize its impact. If they’ll be preparing press materials, please inform our press team as soon as possible -- no later than 48 hours after receiving the formal acceptance. Your manuscript will remain under strict press embargo until 2 pm Eastern Time on the date of publication. For more information, please contact onepress@plos.org.

Kind regards,

David V. Smith, Ph.D.

Academic Editor

PLOS ONE

Additional Editor Comments (optional):

Reviewers' comments:

Reviewer's Responses to Questions

**Comments to the Author**

1. If the authors have adequately addressed your comments raised in a previous round of review and you feel that this manuscript is now acceptable for publication, you may indicate that here to bypass the “Comments to the Author” section, enter your conflict of interest statement in the “Confidential to Editor” section, and submit your "Accept" recommendation.

Reviewer #1: All comments have been addressed

Reviewer #2: All comments have been addressed

**********

2. Is the manuscript technically sound, and do the data support the conclusions?

The manuscript must describe a technically sound piece of scientific research with data that supports the conclusions. Experiments must have been conducted rigorously, with appropriate controls, replication, and sample sizes. The conclusions must be drawn appropriately based on the data presented. 

Reviewer #1: Yes

Reviewer #2: Yes

**********

3. Has the statistical analysis been performed appropriately and rigorously? 

Reviewer #1: Yes

Reviewer #2: Yes

**********

4. Have the authors made all data underlying the findings in their manuscript fully available?

The PLOS Data policy requires authors to make all data underlying the findings described in their manuscript fully available without restriction, with rare exception (please refer to the Data Availability Statement in the manuscript PDF file). The data should be provided as part of the manuscript or its supporting information, or deposited to a public repository. For example, in addition to summary statistics, the data points behind means, medians and variance measures should be available. If there are restrictions on publicly sharing data—e.g. participant privacy or use of data from a third party—those must be specified.

Reviewer #1: Yes

Reviewer #2: Yes

**********

5. Is the manuscript presented in an intelligible fashion and written in standard English?

PLOS ONE does not copyedit accepted manuscripts, so the language in submitted articles must be clear, correct, and unambiguous. Any typographical or grammatical errors should be corrected at revision, so please note any specific errors here.

Reviewer #1: Yes

Reviewer #2: Yes

**********

6. Review Comments to the Author

Please use the space provided to explain your answers to the questions above. You may also include additional comments for the author, including concerns about dual publication, research ethics, or publication ethics. (Please upload your review as an attachment if it exceeds 20,000 characters)

Reviewer #1: (No Response)

Reviewer #2: (No Response)

**********

7. PLOS authors have the option to publish the peer review history of their article (what does this mean?). If published, this will include your full peer review and any attached files.

If you choose “no”, your identity will remain anonymous but your review may still be made public.

**Do you want your identity to be public for this peer review?** For information about this choice, including consent withdrawal, please see our Privacy Policy.

Reviewer #1: No

Reviewer #2: No

# Editor report: Acceptance letter

**DOI:** 10.1371/journal.pone.0262620.r004

**Authors:** David V. Smith

13 Jan 2022

PONE-D-21-31599R1 

Percent framing attenuates the magnitude effect in a preference-matching task of intertemporal choice 

Dear Dr. Anvari:

I'm pleased to inform you that your manuscript has been deemed suitable for publication in PLOS ONE. Congratulations! Your manuscript is now with our production department. 

If your institution or institutions have a press office, please let them know about your upcoming paper now to help maximize its impact. If they'll be preparing press materials, please inform our press team within the next 48 hours. Your manuscript will remain under strict press embargo until 2 pm Eastern Time on the date of publication. For more information please contact onepress@plos.org.

If we can help with anything else, please email us at plosone@plos.org. 

Thank you for submitting your work to PLOS ONE and supporting open access. 

Kind regards, 

PLOS ONE Editorial Office Staff

on behalf of

Dr. David V. Smith 

Academic Editor

PLOS ONE
